# Spatiotemporal dispersion of DENV-1 genotype V in Western Colombia

**DOI:** 10.1093/ve/veaf018

**Published:** 2025-04-16

**Authors:** Diana Rojas-Gallardo, Tyshawn Ferrell, Paula M Escobar-Pereira, Diego Lopez, Beatriz Giraldo, Juliana Restrepo-Chica, Erika Jimenez-Posada, Marlen Martinez-Gutierrez, Julian Ruiz-Sáenz, Autum Key, Nima Shariatzadeh, Dara Khosravi, Megan A Martinez, Andrei Bombin, Jesse J Waggoner, Jorge E Osorio, Christopher J Neufeldt, Matthew H Collins, Jaime A Cardona-Ospina, Anne Piantadosi

**Affiliations:** Population Biology, Ecology and Evolution Graduate Program, Emory University, 201 Dowman Drive, Atlanta, GA 30322, United States; Grupo de Investigación Biomedicina, Facultad de Medicina, Institución Universitaria Visión de las Américas, Avenida de las Américas No 98-56 Sector Belmonte, Pereira, Risaralda 660000, Colombia; Biochemistry, Cell, and Developmental Biology Graduate Program, Emory University, 201 Dowman Drive, Atlanta, GA 30322, United States; Grupo de Investigación Biomedicina, Facultad de Medicina, Institución Universitaria Visión de las Américas, Avenida de las Américas No 98-56 Sector Belmonte, Pereira, Risaralda 660000, Colombia; Grupo de investigación en Inmunología Molecular, Facultad de Ciencias de la salud, Maestría en Ciencias Biomédicas, Universidad del Quindío, Carrera 15 #12N, Armenia, Quindío 63000, Colombia; Grupo de Investigación GIECSA Research Group, Faculty of Health Sciences, Unidad Central del Valle del Cauca (UCEVAValle del Cauca), Carrera 27A, Salida Sur # 48 -144, Tuluá, Valle del Cauca 763021, Colombia; Grupo de Investigación Biomedicina, Facultad de Medicina, Institución Universitaria Visión de las Américas, Avenida de las Américas No 98-56 Sector Belmonte, Pereira, Risaralda 660000, Colombia; Grupo de Investigación GIECSA Research Group, Faculty of Health Sciences, Unidad Central del Valle del Cauca (UCEVAValle del Cauca), Carrera 27A, Salida Sur # 48 -144, Tuluá, Valle del Cauca 763021, Colombia; Grupo de Investigacion en Infecciones Emergentes y Medicina Tropical, Instituto para la investigación en Ciencias Biomédicas—Sci-help, Carrera 37 B 36-05, Torre 1 401, Pereira, Risaralda 660000, Colombia; Grupo de Investigacion en Infecciones Emergentes y Medicina Tropical, Instituto para la investigación en Ciencias Biomédicas—Sci-help, Carrera 37 B 36-05, Torre 1 401, Pereira, Risaralda 660000, Colombia; Grupo de Investigación en Ciencias Animales, Universidad Cooperativa de Colombia, Cl 30 A #33-51, Bucaramanga, Santander 680005, Colombia; Grupo de Investigación en Ciencias Animales, Universidad Cooperativa de Colombia, Cl 30 A #33-51, Bucaramanga, Santander 680005, Colombia; Department of Pathology and Laboratory Medicine, Emory University School of Medicine, 100 Woodruff Circle, Atlanta, GA 30322, United States; Department of Pathology and Laboratory Medicine, Emory University School of Medicine, 100 Woodruff Circle, Atlanta, GA 30322, United States; Department of Pathology and Laboratory Medicine, Emory University School of Medicine, 100 Woodruff Circle, Atlanta, GA 30322, United States; Department of Microbiology and Immunology, Emory University School of Medicine, 100 Woodruff Circle, Atlanta, GA 30322, United States; Division of Infectious Diseases, Department of Medicine, Emory University School of Medicine, 100 Woodruff Circle, Atlanta, GA 30322, United States; Division of Infectious Diseases, Department of Medicine, Emory University School of Medicine, 100 Woodruff Circle, Atlanta, GA 30322, United States; Department of Pathobiological Sciences, School of Veterinary Medicine, University of Wisconsin, 2015 Linden Drive, Madison, WI 53706, United States; Department of Microbiology and Immunology, Emory University School of Medicine, 100 Woodruff Circle, Atlanta, GA 30322, United States; Division of Infectious Diseases, Department of Medicine, Emory University School of Medicine, 100 Woodruff Circle, Atlanta, GA 30322, United States; Grupo de Investigación Biomedicina, Facultad de Medicina, Institución Universitaria Visión de las Américas, Avenida de las Américas No 98-56 Sector Belmonte, Pereira, Risaralda 660000, Colombia; Division of Infectious Diseases and Vaccinology, School of Public Health, University of California, 2121 Berkeley Way, Berkeley, CA 94704, United States; Department of Pathology and Laboratory Medicine, Emory University School of Medicine, 100 Woodruff Circle, Atlanta, GA 30322, United States; Division of Infectious Diseases, Department of Medicine, Emory University School of Medicine, 100 Woodruff Circle, Atlanta, GA 30322, United States

**Keywords:** DENV-1V, spatiotemporal dispersion, phylogenetic analysis, genetic diversity, Colombia

## Abstract

Dengue virus (DENV) is a significant public health concern in Colombia, with increased transmission of DENV type 1 (DENV-1) in the departments of Risaralda and Valle del Cauca in the Central-West region of the country following a large outbreak in 2019. However, little is known about the source, genetic diversity, and evolution of circulating viruses. We obtained serum samples from individuals with acute DENV infection and analysed DENV-1 genetic diversity, phylodynamics, and phylogeography. We found that most viruses belonged to DENV-1 genotype V, and phylogenetic analysis revealed three distinct clades, each of which was most closely related to viruses from neighbouring departments of Colombia sampled over the last 5–10 years. Thus, the 2019 outbreak and subsequent DENV-1 circulation was not due to the introduction of a new lineage to the country but rather reflected local DENV-1 V dispersion and evolution. We identified amino acid positions under positive selection in structural proteins and NS1, which may have a role in immune evasion and pathogenesis. Overall, our analysis of DENV-1 V diversity, evolution, and spread within Colombia highlights the important role of genomic surveillance in understanding virus dynamics during endemic circulation and outbreaks.

## Introduction

Dengue virus (DENV) is estimated to cause 390 million infections globally each year, primarily in tropical and subtropical regions (Guzmán and Kouri 2002, Bhatt et al. 2013, Yang et al. 2021, Nakase et al. 2023). Infection can cause mild symptoms, such as fever, myalgia, and headache, or severe syndromes, including dengue haemorrhagic fever (DHF) and dengue shock syndrome (DSS). DENV belongs to the *Flaviviridae* family and is transmitted by *Aedes aegypti* (primary) and *Aedes albopictus* (secondary) mosquito vectors (Urdaneta-Marquez and Failloux 2011, Rezza 2012). The global distribution of DENV is determined primarily by the geographical range of these mosquito species, but other factors, such as the genetic diversity of the virus, the host population’s immunity, and socio-ecological (i.e. urbanization) conditions, also influence transmission (Muñoz et al. 2021, Ordonez-Sierra et al. 2021).

Dengue poses a significant global public health challenge. Most recent estimates indicate that dengue accounted for 2 922 630 disability-adjusted life years worldwide in 2017, marking a 108% increase since 1990 (Zeng et al. 2021). According to the World Health Organization, in 2023, a total of 4.1 million cases were reported in the Americas including 6710 severe cases and a case fatality rate of 0.05% (World Health Organization 2023). The Pan American Health Organization (PAHO)’s most recent epidemiological update indicates that the total number of cases in the Americas has increased over 20-fold since the 1980s, especially within South America (Gutierrez 2015, Alied et al. 2023). Additionally, DENV-related mortality has increased over 2-fold in the Americas in the past 5 years (Jing and Wang 2019, Pan American Health Organization 2024). However, the extent of DENV’s impact in the Americas is likely not fully appreciated due to limited surveillance.

In addition to diagnostic and epidemiological surveillance, genomic surveillance can be crucial to understanding patterns of dispersion and evolution during both outbreaks and endemic circulation. For example, genomic surveillance can detect new introductions, reveal transmission hotspots, and identify emerging variants with increased transmissibility. This information can, in turn, inform targeted strategies for DENV control. Here, we used genomic epidemiology to investigate DENV transmission and evolution in Western Colombia, which has experienced an increasing burden of disease since 2019.

DENV is a positive-sense RNA virus that has a genome length of 10.7 kb and encodes three structural proteins (capsid, pre-membrane, and envelope) and seven nonstructural proteins (NS1, NS2A, NS2B, NS3, NS4A, and NS5) (Guzman et al. 2010, Thongsripong et al. 2023). DENV comprises four antigenically distinct serotypes (DENV 1-4) whose envelope proteins have an amino acid similarity of only about 70% (Kuno et al. 1998). Within each (sero)type, there are multiple distinct genotypes; viruses share ≤94% genetic similarity between genotypes and ≥95% genetic similarity within genotypes (Rico-Hesse 1990, Weaver and Vasilakis 2009, Gallichotte et al. 2018, Poltep et al. 2021, Yenamandra et al. 2021).

DENV is the most prevalent arbovirus in Colombia and is thought to have been introduced from the Lesser Antilles in the early 1970s (Boshell et al. 1986, Carrillo-Hernandez et al. 2021). DENV-2 was introduced first, followed by DENV-3, DENV-1, and DENV-4 (Padilla et al. 2012). The four DENV types have been co-circulating since the early 1990s, though DENV-1 is currently predominant (Gutierrez-Barbosa et al. 2020). DENV-1, which may be associated with milder symptoms than other types, comprises five genotypes distributed across distinct geographic regions (Cáceres et al. 2008, Villabona-Arenas and Zanotto 2013). Genotype V has been the most predominant in the Americas over the past several decades after replacing genotype III (de Bruycker-nogueira et al. 2016). In addition to endemic circulation, each DENV type has also been reported in region-dependent outbreaks across the country (Ramos-Castañeda et al. 2017). Colombia has six regions: Andean (Central), Amazonia, Pacific Coast, Orinoco, Caribbean Coast, and Insular Region. The Central-West region (encompassing the Andean) has had five major DENV outbreaks: in 1989, 2002, 2010, 2013, and 2019 (Padilla et al. 2012).

In 2019, starting from the eighth epidemiological week, Colombia experienced a new epidemic phase of dengue in which the reported cases were above the upper limit compared to its historical behaviour (2011–18) and the accumulated incidence was 465.9 cases per 100 000 residents at the end of the year (Reyes et al. 2019). Since then, the number of DENV cases in Colombia has nearly doubled, and severe dengue increased over 2-fold in 2023 compared to 2019 (Pan American Health Organization, 2024).

We hypothesized that viral factors may have contributed to this increased disease burden, for example the introduction of new lineages and/or mutations that conferred immune evasion. Prior studies have highlighted a role for clade replacement in driving DENV outbreaks in other locations (OhAinle et al. 2011, Lambrechts et al. 2012, O’Connor et al. 2021), and Salvo *et al*. described both serotype shift (from DENV-1 to DENV-3) and within-DENV-1 lineage replacement in the Colombian department of Antioquia from 2014 to 2016 (Laiton-Donato et al. 2019, Salvo et al. 2019). However, DENV transmission dynamics and evolution in the Central-West region of Colombia remain understudied, despite the high incidence of DENV in this region.

We generated new DENV surveillance and sequence data from 2019 to 2022 to investigate the origin, genetic diversity, and spatiotemporal dispersion of DENV lineages across departments in Colombia. We particularly focused on two nearby cities in the Central-West region with different disease burdens, Cali located in Valle del Cauca (303 cases per 100 000 residents in 2021) and La Virginia located in Risaralda (18 cases per 100 000 residents in 2021) (**Figure S1**). We focused on DENV-1 genotype V (DENV-1 V), which was the most common virus identified in our contemporary sequences, and which has previously been understudied, with only about 30 full-length sequences from Colombia published in GenBank since 1988. Prior to the samples collected in our study, only one DENV genome sequence had been generated from the departments of Risaralda and Valle del Cauca, underscoring the need for increased genomic surveillance in this region. Our new sequence data allowed us to investigate patterns of dispersion and evolution within the Central-West region and place this in the context of DENV dynamics within the country and region.

## Methods

### Ethics statement

The acute febrile illness study in La Virginia, Risaralda, was approved by the ethics committee of the Universidad Cooperativa de Colombia and by the Emory University Institutional Review Board (STUDY# 00004665). Samples collected in Cali, Valle del Cauca, were remnant clinical specimens, and this study has the approval of the ethics committee of the Universidad Unidad Central del Valle del Cauca (UCEVA). Work performed with these samples at Emory University was considered secondary use of deidentified samples, and no IRB review was required.

### Study locations, including DENV burden

Colombia is administratively divided into 32 departments (first administrative level). In accordance with the guidelines of the National Public Health Surveillance System, all probable and confirmed cases of dengue require mandatory reporting to the National Public Health Surveillance System (SIGIVILA). This process includes data consolidation and report from the local institution to the departmental institution, and then analysis and publication by the National Health Institute [Instituto Nacional de Salud (INS)].

The municipality of La Virginia belongs to the department of Risaralda in the central-west part of Colombia and had a total population of 28 488 as of 2023. La Virginia, referred to by its department Risaralda hereafter, is located approximately 30 km from the state capital, Pereira. It is 200 km north of Cali, the capital of the department of Valle del Cauca, and 210 km south of Medellín, the capital of the department of Antioquia. Risaralda has endemic DENV circulation and reported 153 cases in 2019, 125 cases in 2020, 26 cases in 2021, and 22 cases in 2022 to SIVIGILA (SIVIGILA, consulted in August 2023).

The city of Cali, located in southwestern Colombia, is the third largest city in the country with a population of 2 280 522 as of 2023. Cali, referred to by its department Valle del Cauca hereafter, has endemic circulation of dengue virus with seasonal outbreaks and reported a total of 3889 cases in 2019 (3.1% of the national total), 13 232 cases in 2020 (16.9% of the national total), 5726 cases in 2021 (11% of the national total), and 2840 cases in 2022 (4.1% of the national total) (data from the epidemiological bulletins, INS).

### Sample collection

Serum samples were collected from patients in a prospective cohort study in Risaralda between July 2019 and April 2022. In this cohort, patients residing in the Central-Western Metropolitan Area of Risaralda were recruited at the San Pedro y San Pablo Hospital if they presented with a febrile condition that was diagnosed as probable dengue. A trained nurse was responsible for informed consent and assent, verifying the inclusion and exclusion criteria. Clinical assessment and classification were performed by the attending physician, and data were obtained from clinical records and a structured questionnaire.

Serum samples from patients in Valle del Cauca were obtained from August 2021 to December 2022 at a medical centre located north of the city. These were residual clinical samples from patients diagnosed with dengue by clinical laboratory testing.

### DENV-1 detection and typing

Samples from both Risaralda and Valle del Cauca were transported to the molecular biology laboratory at the Universidad Vision de las Américas, and a rapid test was used to detect the NS1 antigen and IgG and IgM envelope-specific antibodies (Bioline™ Dengue Duo). RNA was extracted from samples positive for NS1 and IgM using the PureLink™ RNA Mini Kit (Invitrogen). To confirm dengue infection and detect infections with other arboviruses, such as Zika virus and chikungunya virus, a multiplex reverse transcription quantitative polymerase chain reaction (RT-qPCR) assay was performed using a previously described assay (Waggoner et al. 2016). PCR results were confirmed for all samples by testing a duplicate aliquot using the same assay at Emory University. In RT-qPCR positive samples, the DENV serotype was determined by a multiplex real-time RT-qPCR method as previously described (Waggoner et al. 2013).

### Full DENV genome sequencing

We used two approaches to generate full-length DENV genome sequences: metagenomic and multiplex amplicon sequencing. For both approaches, we first performed heat-labile dsDNase treatment (ArcticZymes, Tromso, Norway) and first-strand cDNA synthesis with SUPERSCRIPT IV RT and random primers (Fisher/Invitrogen, Waltham, MA, USA).

### Metagenomic sequencing and analysis

For metagenomic sequencing, second-strand cDNA synthesis was performed using *Escherichia coli* DNA ligase, polymerase I, RNase H, dNTP mix, and Second Strand Buffer (NEB, Ipswich, MA, USA). Double-stranded cDNA underwent Nextera XT library construction (Illumina, San Diego, CA, USA) and Illumina sequencing as previously described (Langsjoen et al. 2023). As a positive control, External RNA Controls Consortium (ERCC) spike-ins (Invitrogen, Waltham, MA, USA) were added prior to cDNA synthesis. The ERCC spike-ins also served as a unique control for each sample to measure cross-contamination. At least one negative control (water) was included with each library construction batch. After Illumina sequencing, adapter trimming was conducted in BaseSpace using bcl2fastq2 v2.20 to mask short adapter reads (*n* = 35) and trim to a minimum read length (*n* = 35). Reads were processed using viral-NGS v1.25.0 (Park 2019) in the following manner: they were converted to unmapped bam files, quality control checked for the correct ERCC spike-in, and taxonomically classified using KrakenUniq. Reads underwent reference-based assembly, initially using the DENV-1 RefSeq sequence (NC_001477). The resulting partial genomes were evaluated by BLAST to find the best-matching reference sequence, OM654348, which was used for final reference-based assembly, all using viral-NGS v1.25.0 (Park 2019).

### Multiplex amplicon sequencing primer design

We designed a new multiplex amplicon sequencing protocol to specifically sequence lower-concentration DENV-1 genotype V viruses from this study based on the sequence data we had generated by metagenomic sequencing (**Figure S2**) (Su et al. 2022). Multiplex amplicon primers were designed using a multiple sequence alignment of five DENV-1 Genotype V sequences we had generated by metagenomic sequencing, which had coverage of >99% and mean depth of >200× and represented different departments of Colombia. PrimalScheme v1.4.1 (Quick et al. 2017) was used to generate 34 primer pairs that amplified ∼400 nucleotide (nt) overlapping fragments spanning the entire genome. These primers were mapped to our sequences to check for mismatches and amplicon lengths. Any primers that had multiple binding sites or could lead to primer-dimer formation were modified. Primers with a Tm above 50.1°C (ranging from 55.9°C to 61°C) were selected to reduce self-dimer and hairpin secondary structures. The amplicon primers from PrimalScheme were concatenated with Nextera XT indexes to create fusion primers, which contained a forward iNEXT universal primer (5ʹ TCGTCGGCAGCGTCAGATGTGTATAAGAGACAG 3ʹ) at the 5ʹ end of the forward primer and a reverse iNEXT universal primer (5ʹ GTCTCGTGGGCTCGGAGATGTGTATAAGAGACAG 3ʹ) at the 5ʹ end of the reverse primer, as shown in (**Table S1**) (Glenn et al. 2019). The fusion primers were synthesized by Integrated DNA Technologies (Iowa, USA).

### Multiplex amplicon library construction and sequencing

To generate DENV-1 amplicons from the first-strand cDNA template described above, the DENV-specific multiplex primers were separated into two pools (odd and even numbers from **Table S1**), and multiplex PCR was performed in two separate reactions per sample to reduce the overextension of neighbouring amplicons. Each multiplex PCR contained cDNA template (3 µl), Q5 Hot Start high-fidelity DNA polymerase (12.5 µl) (NEB, Ipswich, MA, USA), nuclease-free water (7.5 µl), and the odd or even primer pool (2 µl). The multiplex PCR cycle parameters were set as follows: 98°C for 30 s, 30 or 34 cycles at 95°C for 15 s, and 65°C for 5 min. The cycle number depended on the DENV C_T_ value: 30 cycles were used for samples with a C_T_ value ≤ 30, while 33 cycles were used for samples with a C_T_ value above 30. The amplicons underwent a 13-cycle Nextera indexing PCR (Illumina, San Diego, CA, USA) using dual unique indexes. Resulting libraries were quantified using the KAPA Universal Complete Kit (Roche, Basel, Switzerland), pooled to equimolar concentration, and sequenced using a Miseq 600 cycle v3 kit (Illumina, San Diego, CA, USA) with paired-end 300bp reads. The median number of reads per sample was 3.03M (range: 882 K–8.47M).

### Reference-based assembly

FASTQ files from multiplex amplicon sequencing were analysed using ViralRecon v2.6.0 (Patel 2023). Briefly, reads were filtered based on the Q30 score, length (minimum 50 bp), and Qphred score (minimum 30) using FastQC v0.11.9. The reads underwent adapter and quality trimming before removing host reads using Fastp v0.23.2 and Kraken 2 v2.1.2. Next, the paired-end reads were mapped to the reference sequence (OM654348) using Bowtie 2 v2.4.4. Consensus sequences were then assembled in ViralRecon.

### Optimizing multiplex amplicon primer pooling

To optimize the concentration of each primer in our multiplex amplicon pools, we first sequenced three samples that had previously been sequenced using the metagenomic approach, measured the per-amplicon depth using mosdepth v0.3.3, and calculated a sequence read fraction (SRF) by dividing the amplicon’s depth by the median depth across the whole genome. Amplicons with an SRF value between 0.75 and 1.3 were deemed adequate. For amplicons with SRF values lower or higher than this range, we increased or decreased the primer concentrations, respectively, and repeated testing. The final primer concentrations are listed in **Table S1**.

### Alignment with reference sequences

All DENV-1 V full genome and envelope coding sequences were downloaded from National Center for Biotechnology Information (NCBI), and their genotypes were verified using Genome Detective v3.83 (Fonseca et al. 2019, Vilsker et al. 2019). We constructed separate alignments for the full coding region (CDS) and the envelope coding region, including our 24 newly generated sequences, as well as 996 available CDS reference sequences, and 1382 available envelope reference sequences (which include both the envelope portion of the CDS sequences and envelope-only sequences). Alignments were generated using the MAFFT v7.490 plugin in Geneious Prime. These full datasets were used to construct maximum likelihood (ML) phylogenetic trees, as described below. Supplementary file one contains a list of all GenBank sequences that were included in each dataset.

We then used two subsampling schemes to create smaller datasets amenable to complex inference models (Mongiardino Koch and Satta 2021). For the first subsampling scheme, which was based on genetic proximity, we used the jukes-cantor model to create a pairwise genetic distance matrix, and we selected 30 reference sequences for each of our newly generated sequences based on those with the closest genetic proximity; after initial analyses, we further restricted this to the smallest clade that contained all of our newly generated sequences. For the second subsampling scheme, which was based on geography, we included up to 10 unique sequences from each country in the Americas per year. In both schemes, we retained all sequences from Colombia. Thus, our final subsampled alignments were: (I) CDS with genetic proximity subsampling (83 DENV-1 V sequences from Colombia and 126 DENV-1 V sequences from other locations); (II) envelope with genetic proximity subsampling (163 DENV-1 V sequences from Colombia and 137 DENV-1 V sequences from other locations); (III) envelope with genetic proximity subsampling, restricted to the smallest clade that contained all of our newly generated sequences (94 DENV-1 V sequences from Colombia and 53 DENV-1 V sequences from other locations) and (IV) envelope with geographic subsampling (163 DENV-1 V sequences from Colombia and 435 sequences from elsewhere in the Americas) (Supplementary file one). We did not analyse a CDS geographically subsampled dataset, as we found it was important to include reference sequences for which only the envelope region was available, as described in the ‘Results’ section.

### Phylogenetic analysis

We evaluated potential recombination using Phitest and the genetic algorithm for recombination detection (GARD) (Kosakovsky Pond et al. 2006, Huson et al. 2008), and did not find any evidence for recombination in either the CDS or envelope dataset.

ML trees were initially constructed from the CDS and envelope datasets (prior to subsampling) using IQ-TREE v1.6.12 (Nguyen et al. 2015) with the following parameters: 1300 ultrafast bootstraps and 1000 replicates for the SH-like approximate likelihood ratio test. The best-fit model for the CDS dataset was GTR + F + G4, as determined by the Bayesian Information Criterion using the ModelFinder software, and the gamma shape alpha was 0.263 (Kalyaanamoorthy et al. 2017). The best-fit model for the envelope dataset was TN + F + I + G4, and the gamma shape alpha for the envelope ML tree was 1.292. The ML trees were also generated for each subsampled dataset using IQ-TREE and ModelFinder. The best-fit models for the CDS genetic proximity-subsampled, envelope genetic proximity-subsampled, and envelope geography-subsampled datasets were TIM2 + F + I + R2, TN + F + I + G4 (Gamma shape alpha: 0.97), and TN + F + R4, respectively. The ML trees were visualized in iTOL (Letunic and Bork 2021) and rooted to the midpoint.

### Molecular clock and phylogeographic inference

To investigate the spatiotemporal dynamics of DENV-1 V, we first measured temporal signal using TempEST (Rambaut et al. 2016), which evaluates the correlation between sampling dates and genetic distance through root-to-tip regression. All subsampled datasets demonstrated a strong correlation coefficient (>0.75), suggesting sufficient temporal signal for time-scaled phylogenetic analysis.

To identify the optimal molecular clock and population coalescent models, we used a nested sampling (NS) scheme to compare the Strict and Relaxed Log Normal clocks, as well as the Constant, Exponential, Bayesian Skyline, and Extended Bayesian Skyline population models using BEAST v2.6.7, BEAUti v2.6.7, and the NS v1.1.0 package (Bouckaert et al. 2019). For the CDS genetic proximity-subsampled dataset (1 above), a run of 70 particles with 60 000 chainLength was required to determine the best-fitting model; for the two envelope genetic proximity-subsampled datasets (2 and 3 above), runs of 16 particles with 20 000 chainLength was sufficient; and for the envelope geography-subsampled dataset (4 above), a run of 24 particles with a chainLength of 20 000 was used. The results of these runs were analysed using NSLogAnalyser in BEAUti v2.6.7.

Phylogeographic reconstruction was performed using the discrete trait model in the BEAST_CLASSIC v1.5.0 package. Due to the complexity and quantity of the location traits to be evaluated (at the level of countries around the globe and departments within Colombia) and to optimize run times, computational resources, and avoid over-parameterization of the models, the phylogeographic analysis was set as follows.

For the genetic proximity-subsampled datasets (full CDS and envelope, datasets 1 and 2 above), the location traits were set by country. For the envelope genetic proximity-subsampled dataset restricted to the smallest clade that contained our sequences (dataset 3 above), the location traits were set as departments for Colombian sequences and country for the rest. Finally, for the envelope geography-subsampled dataset (dataset 4 above), the location trait was set to department (for Colombian sequences), to country for neighbouring countries (Brazil, Peru, Ecuador, and Venezuela), and division for others (South America, Central America, Caribbean, and North America).

We used the optimal molecular clock and population growth models identified above. For the CDS genetic proximity-subsampled dataset, 500 million chains were run, while for the two envelope genetic proximity-subsampled datasets, 300 million chains were run, and for the envelope geography-subsampled dataset, 900 million chains were run. BEAGLE was used to improve run performance (Ayres et al. 2012). Tracer v1.7.1 (Rambaut et al. 2018) was used to visualize parameter distributions and traces for the posteriors, and to verify that ESS values were higher than 300. For each dataset, a maximum clade credibility (MCC) tree was summarized using the program TreeAnnotator v2.6.2 with a burn-in percentage of 10. The trees were visualized in iTOL (Letunic and Bork 2021).

### Selection analysis

We used HyPhy to evaluate both pervasive and episodic selection (Kosakovsky Pond et al. 2020). We used pervasive selection models SLAC (Single-Likelihood Ancestor Counting), FEL (Fixed Effects likelihood), and FUBAR (Fast, Unconstrained Bayesian AppRoximation), as well as episodic selection model MEME (Mixed Effects Model of Evolution) to analyse the CDS genetic proximity-subsampled dataset and the envelope genetic proximity-subsampled dataset. We used the aBSREL (adaptive Branch-Site Random Effects Likelihood) model to identify specific branches under selection.

To characterize amino acid changes within our sequences, we reconstructed their most recent common ancestor (MRCA) using TreeTime (Sagulenko et al. 2018) and identified nonsynonymous substitutions compared to this MRCA using Geneious Prime v3.2.1.

## Results

### Molecular testing demonstrates predominance of DENV-1 in Western Colombia

Between July 2019 and April 2022, a total of 178 febrile patients were recruited in our study site in Risaralda. Among them, 20 DENV infections were confirmed through detection of the NS1 antigen, IgM envelope-specific antibodies, and DENV RNA by RT-qPCR (**Table S2**). The median age of confirmed cases was 9 years, with 42% of patients falling between 5- and 15-year-olds. Males accounted for 52.6% of the cases, and only one participant reported a previous DENV diagnosis. These cases accounted for 8.2% of the DENV infections reported by the national surveillance system for Risaralda during the same study period. In addition, between August 2021 and November 2022, 201 samples were collected from patients clinically diagnosed with DENV in Valle del Cauca (**Figure S1**). A total of 96 of these samples were positive by the NS1/IgM rapid test, 79 of which were positive for DENV RNA by RT-qPCR (**Table S2**). The median age of these confirmed cases was 12 years with 73% of patients falling between 5- and 15-year-olds; 59.5% were females. No infections or coinfections with other arboviruses were identified in patients at either location based on RT-qPCR assay testing for DENV, chikungunya virus, Zika virus, and Mayaro virus.

To identify circulating serotypes, we performed type-specific RT-qPCR. Among the 20 DENV RNA-positive samples from Risaralda, 8 had sufficient DENV RNA concentration for serotype determination; 7 were DENV-1 and 1 was DENV-2. Among the 79 DENV RNA-positive samples from Valle del Cauca, 66 had sufficient DENV RNA for type determination; 42 were DENV-1, 20 were DENV-2, and 4 were DENV-3. Given that DENV-1 was the most prevalent type, we focused our sequencing and phylogenetic analysis on DENV-1. We obtained full-length DENV-1 sequences for seven samples from Risaralda (from 2019 to 2022) and 17 samples from Valle del Cauca (from 2021 to 2022) (**Table S3**) using a combination of metagenomic sequencing and multiplex amplicon sequencing.

### Phylogenetic analysis reveals unique regional clades of DENV-1 Genotype V

**Figure 1.**
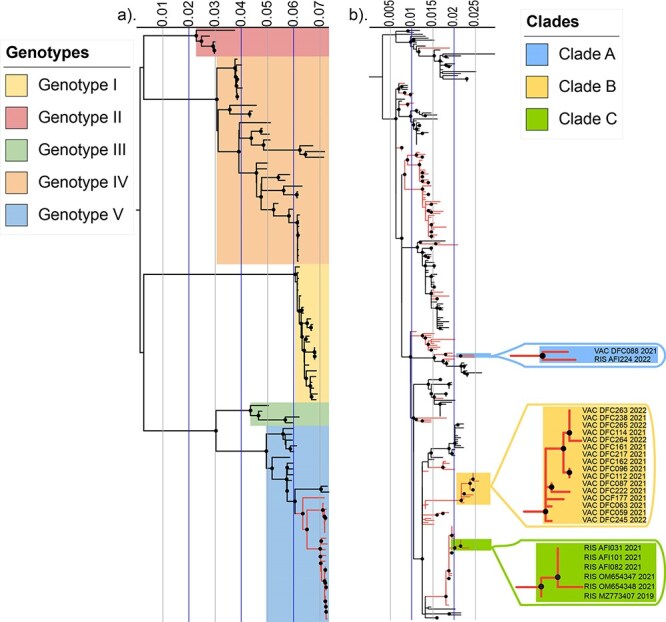
ML phylogenetic analyses of 24 newly generated DENV-1V sequences compared to reference sequences. (a) The full-length coding regions for the 24 newly generated sequences in this study were aligned with 79 reference sequences representing each DENV-1 genotype, and underwent ML phylogenetic analysis. Red branches indicate the 24 newly generated sequences in this study. (b) The envelope coding regions for the 24 newly generated sequences in this study were aligned with 276 reference sequences that had been selected by genetic proximity (subsampled dataset 2 in ‘Methods’ section), and underwent ML phylogenetic analysis. Red branches indicate all sequences from Colombia. The 24 newly generated sequences in this study clustered in three distinct clades, Clade A (blue), Clade B (yellow), and Clade C (green). For both trees, black circles indicate nodes with an ultrafast bootstrap value greater than or equal to 95, and the internal tree scale represents the nucleotide substitutions per site.

We sought to define the genetic diversity of DENV-1 in Colombia using our 24 newly generated sequences as well as publicly available sequences. All of our DENV-1 sequences were classified as genotype V by the DENV genotyping tool (Vilsker et al. 2019), and this was confirmed in our initial phylogenetic analysis (**Fig. 1a**). According to the new classification proposed by Hill *et al*. (Hill et al. 2024), 22 of our sequences were classified as dengue virus serotype 1, genotype V, lineage D.1 (DENV-1 V_D.1) and two sequences were classified as dengue virus serotype 1, genotype V, lineage D.2 (DENV-1 V_D.2).

To further explore their phylogenetic relationships, we constructed a ML tree using all 996 available full CDS DENV-1 V reference sequences from NCBI and our 24 new sequences (**Figure S3**) and subsampled this dataset by genetic proximity to include 209 total sequences (including our new 24) in order to facilitate further analysis and visualization (**Figure S4**). The resulting phylogenetic trees demonstrated that our sequences clustered into three distinct clades. We repeated this analysis using the envelope coding sequence only because there are a larger number of available envelope reference sequences (1382 total, including those represented in the CDS dataset, **Figure S5**), capturing greater geographic and temporal diversity than the available full-length sequences. We again used genetic proximity subsampling to select 300 total sequences (including our new 24) for ease of visualization and further analysis (**Fig. 1b**).

Analysis of the envelope coding sequence confirmed that the sequences generated in this study grouped into three distinct clades and further demonstrated that each clade was most closely related to other sequences from Colombia (**Fig. 1b**). Clade A, within DENV-1 V_D.2, contained one sequence from Risaralda in 2022 and one sequence from Valle del Cauca in 2021 (**Fig. 1b**, blue highlight), which clustered together with a reference sequence obtained in 2015 from Cundinamarca, a department in central Colombia (**Figure S1**). Clade B contained 16 DENV-1 V_D.1 sequences from Valle del Cauca in 2021–22 (**Fig. 1b**, yellow highlight), which clustered with sequences from Santander, a department located in the central-northern region of Colombia (**Figure S1**). Clade C contained six DENV-1 V_D.1 sequences from Risaralda in 2019–21 (**Fig. 1b**, green highlight), which clustered with sequences obtained in 2016 from Antioquia, an adjacent department located in the northwest of Colombia (**Figure S1**).

Overall, analysis of the envelope coding sequences provided more information compared to the full genome sequences, since it allowed us to include closely related viruses for which only the envelope coding sequences were available. For example, the relationship between the sequences in Clade B and sequences from Santander would not have been apparent from the analysis of only full genome sequences (**Figure S3**). Together, these data demonstrate the circulation of three unique clades of DENV-1 V_D circulating at the same time in geographically distinct, but nearby, regions of Colombia.

### Phylodynamic and phylogeographic analyses reveal persistence, evolution, and local transmission of DENV-1 clades in western Colombia

Analysing the genetic diversity of DENV-1 across temporal and spatial scales can be used to understand how specific lineages are introduced, spread, and maintained in specific regions. To perform phylodynamic analyses, we formally compared molecular clock and population growth models for both the CDS and envelope datasets. For the CDS genetic proximity-subsampled dataset, the best-fit model used a relaxed lognormal clock with a Bayesian skyline demographic model. For the two envelope genetic proximity-subsampled datasets, a strict molecular clock with a Bayesian skyline demographic model was the best fit, though a relaxed lognormal clock with a Bayesian skyline demographic model had a very similar likelihood (**Table S4**). For the envelope geography-subsampled dataset, a strict molecular clock with a Bayesian skyline demographic model was the best fit. The MRCA dates for the two datasets were similar for the nodes of interest, and the confidence intervals were comparable (**Table S5**).

Phylogeographic analysis of the envelope genetic proximity-subsampled dataset allowed us to evaluate the dispersion of DENV-1 between Colombia and other countries (**Figure S6**). We observed repeated exchange of DENV-1 between the Colombian departments of Santander and Norte de Santander and the neighbouring country of Venezuela until the 2000s. After the 2000s, we observed only two introductions to Colombia, one from Venezuela [location probability (LP) = 0.97] introduced into the department of Antioquia in August 2009, and another from Ecuador (LP = 1), introduced into the department of Cauca in February 2012. On the other hand, two dispersions to other countries with a high probability of ancestral location in Colombia were identified, one to Peru in July 2015 (LP = 1) and another to Ecuador in August 2007 (LP = 0.99). A third introduction to Venezuela was also identified but in this case with a probability of ancestral location in Colombia of LP = 0.46 and a probability of ancestral location in Venezuela of LP = 0.34. Additionally, this global analysis by country showed that the newly generated sequences have common ancestors with sequences previously identified in Colombia. Analysis of the CDS genetic proximity-subsampled dataset (**Figure S7**) also showed similar results for both the dispersion between countries and for an ancestral location in Colombia of the sequences generated in this study.

**Figure 2.**
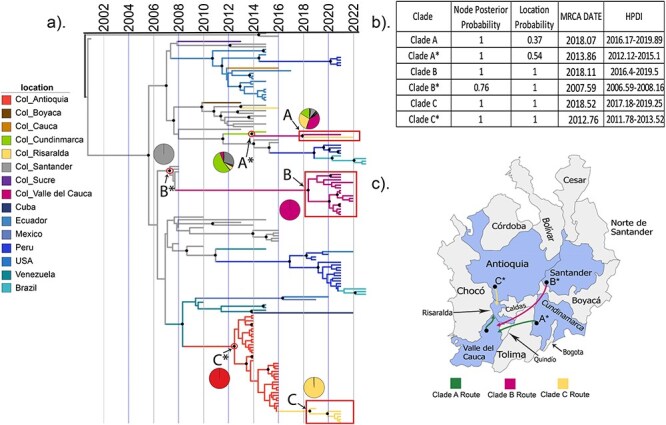
Temporal and geographical analysis of 24 newly generated DENV-1V envelope sequences. (a) The envelope coding regions for the 24 newly generated sequences in this study were aligned with 123 reference sequences from the Americas that had been selected by genetic proximity (subsampled dataset 3 in ‘Methods’ section) and underwent Bayesian analysis. On a time-scaled MCC tree, the 24 newly generated sequences in this study clustered in three clades, which are highlighted with red boxes. Black circles indicate nodes with a posterior probability of 90% or greater. Branches are coloured by location or inferred location, and pie charts indicate the posterior LP for each node of interest. Colombian sequences are coloured with department-level resolution; location names are Col_ followed by the department name. (b) Posterior probability of the node position, location, and date, including 95% highest posterior density (HPD), for the labelled nodes. (c) Most probable dispersion routes of clades identified in this study.

To evaluate the dispersion of DENV-1 V across Colombia, we used the envelope genetic proximity-subsampled dataset that was restricted to the smallest clade containing our newly generated sequences (**Fig. 2**). This allowed us to obtain resolution at the department level within Colombia. Overall, we found that each of Clades A-C was introduced to Risaralda and Valle del Cauca from neighbouring departments within Colombia. Our results suggest that these clades and their immediate ancestors have been circulating and evolving within Colombia within the last 15 years. For example, the summarized MCC tree (**Fig. 2a**) estimates the time of the MRCA (tMRCA) of Clade A sequences as January 2018 (HPDI = February 2016—November 2019, **Fig. 2b**). Although the Clade A MRCA was within Colombia, its specific location is uncertain, with a LP of 0.37 for Valle del Cauca, 0.29 for Risaralda, and 0.16 for Cundinamarca. The closest relative to Clade A was a sequence obtained from Cundinamarca in 2015. Clade A and this sequence share an MRCA, referred to as A*, that circulated around 2013 (HPDI = February 2012—February 2015) and whose location was Cundinamarca with a probability of 0.54. Cundinamarca is a department located in the centre of Colombia, whose capital is approximately 300 km from the capital of Risaralda and approximately 450 km from the capital of Valle del Cauca (**Fig. 2c**, **Figure S1**).

The tMRCA of Clade B sequences was February 2018 (HPDI = May 2016—July 2019). Clade B was most closely related to sequences from Santander, whose MRCA (B*) dates to approximately August 2007 (HPDI = July 2006–January 2008) with a location in the department of Santander (LP = 0.99). The capital of Santander is located 556 km from the capital of Risaralda and 760 km from the capital of Valle del Cauca (**Figure S1)**.

The tMRCA of Clade C sequences was July 2018 (HPDI = March 2017–April 2019). This clade was most closely related to sequences from Antioquia, whose MRCA (C*) dates to October 2012 (HPDI= October 2011–July 2013) with a location in Antioquia (LP = 1.0). The capital of Antioquia is located approximately 250 km from the capital of Risaralda and approximately 450 km from the capital of Valle del Cauca (**Figure S1)**.

Analysis of the larger envelope genetic proximity-subsampled dataset (**Figure S7**) and the envelope geography-subsampled dataset (**Figure S8**), which included 435 reference sequences from outside Colombia, showed similar results. Thus, overall, these results indicate that each of the three contemporaneous DENV-1 V clades in western Colombia was derived from viruses circulating in nearby regions of Colombia within the last 15 years. Based on these data, the increase in DENV cases observed since 2019 was not due to the introduction of new virus lineages, though the long branches on our phylogenetic tree (especially leading to Clade B) highlight a genomic surveillance gap in DENV circulation and evolution in this region.

### Clade-specific amino acid substitutions occurred across the genome

**Figure 3.**
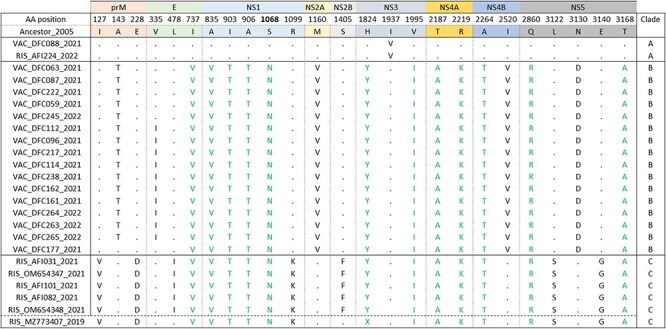
Amino acid changes observed in 24 newly generated DENV-1V sequences, compared to the 2005 ancestral sequence. Upper rows indicate the protein region and specific amino acid position within the DENV polyprotein where each substitution occurred. The substitutions in black font are clade-specific, while those in green font are found in multiple clades. Dots represent conserved positions. The horizontal dashed line at the bottom separates the Risaralda sequence found in 2019 from those found in 2021.

To evaluate DENV-1 genetic changes that may be associated with recently increased disease burden, we investigated amino acid substitutions and sites under positive selection in contemporary viruses. We identified 25 nonsynonymous substitutions in clades A (*n* = 1), B (*n* = 16), and C (*n* = 18) compared to the ancestor of DENV-1 V sequences from Colombia (**Fig. 3**). The highest numbers of substitutions were found in the NS5 (*n* = 5) and NS1 (*n* = 5) proteins. Clade A had only one clade-specific substitution in the NS3 protein and did not share substitutions with the other clades. Clades B and C shared 12 substitutions with one another. Clade B also had 5 clade-specific substitutions across the prM, envelope, NS2A, NS4B, and NS5 proteins. Clade C had 7 clade-specific substitutions across the prM, envelope, NS1, NS2B, and NS5 protein, though L478I (envelope) and S1405F (NS2B) were only present in sequences from 2021. Thus, DENV-1 V viruses circulating contemporaneously in nearby locations were distinct at the amino acid level, including potentially antigenic regions.

We performed selection analysis using a combination of pervasive and episodic models in HyPhy (Kosakovsky Pond et al. 2020) and identified two codons with a high probability of positive selection (**Table S6**). First, in the CDS proximity-subsampled dataset, position 293 in the NS1 coding region (corresponding to position 1068 in the full CDS) was identified by MEME, FUBAR, FEL, and SLAC models (p-value < .05, posterior probability of positive selection/PPPS = 0.99) as being under positive selection. Our Clades B and C shared substitution S293N at this site compared to ancestral sequences; however, our adaptive branch-site model (aBSREL) analysis did not have enough resolution to definitively say that the branches for Clades B and C were under positive selection. Second, in the envelope proximity-subsampled dataset, position 248 within the E coding region was identified by FEL, FUBAR, SLAC, and MEME models (p-value <0.07, PPPS = 1) as being under positive selection. The specific branches under positive selection did not include our sequences or any other sequences from Colombia. This position was not identified as being under positive selection in the CDS dataset because the branches under positive selection were derived from envelope-only sequences, not complete genome sequences.

## Discussion

DENV genomic surveillance is crucial to understanding virus evolution and transmission in endemic locations and during outbreaks. We investigated recent DENV-1 diversity and circulation in Central-West Colombia, a region with very little prior genomic surveillance, during a time of increased disease burden. We detected three distinct clades of DENV-1 V: one in Risaralda, one in Valle del Cauca, and one that co-circulated in both departments. We found that each clade had been circulating in nearby departments of Colombia over the preceding 15 years, and thus the increased disease burden observed since 2019 did not reflect introduction of new lineages. Although a number of prior studies have linked DENV outbreaks with clade replacement, our results add to the mounting evidence that longstanding virus lineages within a population can contribute to increased disease burden, likely due to shifting environmental factors or demographics (Ashall et al. 2023, Martínez et al. 2024).

Interestingly, the new 2019–22 DENV-1 V sequences from Risaralda were not related to the prior DENV-1 V sequence reported from that department in 2017, which instead clustered with sequences from Santander. Thus, there appear to have been at least three introductions of distinct DENV-1 V clades into Risaralda from different departments to the northeast (Santander, Antioquia, and Valle del Cauca) over the preceding 5 years. We observed similar results for Valle del Cauca, with introductions likely from Santander and Cundinamarca sometime in the last 15 years. Notably, the long branch lengths between our clade MRCAs and their ancestors (especially B and B*) reflect unmeasured circulation and underscore the need for increased genomic surveillance. Because of this, our results must be interpreted with caution, especially since the lack of sequence data from southern and eastern regions of Colombia may mask patterns of transmission and evolution.

On a larger scale, DENV-1 V has a wide spatiotemporal dispersion in Colombia and other South American countries and has been the predominant genotype of DENV-1 in the Americas since its introduction (de Bruycker-nogueira et al. 2016, Jiménez-Silva et al. 2018). DENV-1 V was introduced into the Americas from India twice, once in the early 1970s and a second time in the 1990s (Villabona-Arenas and Zanotto 2013, Walimbe et al. 2014). The earliest DENV-1 V cases were reported in the Caribbean islands and later spread to South America, becoming endemic in many countries (Walimbe et al. 2014, Ribeiro et al. 2021). However, in the 1990s, most of the original DENV-1 V Caribbean lineages became extinct and were replaced by a lineage that had evolved in Venezuela and another lineage that had evolved in Brazil (de Bruycker-nogueira et al. 2016). Here, we observed repeated exchange of DENV-1 V between the Colombian departments of Santander and Norte de Santander and Venezuela, which is consistent with what has been previously reported (Jiménez-Silva et al. 2018, Carrillo-Hernandez et al. 2021). We also observed dispersion of DENV-1 V from Colombia to Ecuador, consistent with a prior study suggesting that Colombia and Venezuela are the main origins of DENV-1 dissemination to Ecuador (Maljkovic Berry et al. 2020). However, this dispersion is not unidirectional since we also identified a strain in the southwestern department of Cauca with common ancestry to clades reported in Ecuador in 2011. Interestingly, sequences recently sampled in Peru (2021) have an MRCA in Colombia with circulation in September 2018, which demonstrates active exchange of DENV between the southwest region of Colombia and neighbouring countries. These results highlight Colombia’s role not only as a recipient but also as an exporter of DENV cases. Strengthening of genomic surveillance in these regions is needed to understand how the countries on the southern borders contribute to the exchange of lineages within Colombia.

Similar to our results, prior studies have also demonstrated the importance of local evolution of DENV in Colombia. Laiton-Donato et al. reported the divergence of two DENV2 strains with different geographical distributions between 2013 and 2016 (Laiton-Donato et al. 2019). Salvo *et al*. reported DENV-1 clade replacement during an interepidemic season (2014–16) in a single location (Medellín, Antioquia) (Salvo et al. 2019). In both examples, virus spread was potentially linked to amino acid substitutions, within the E protein in the first report and NS1 in the second, suggesting that specific virus adaptations may facilitate transmission.

Here, we identified clade-specific amino acid mutations in contemporary DENV-1 sequences from Colombia, including three mutations in prM. The prM protein forms a heterodimer with the envelope protein, ensuring proper folding and virus particle assembly while preventing premature fusion within intracellular membrane compartments (Wang et al. 2009). In a recent study, anti-prM antibodies were observed to increase pathogenesis in a mouse model and increase the cell entry of partially mature viruses, which otherwise have reduced entry compared to mature viruses (Dejnirattisai et al. 2010, Dowd et al. 2023). Thus, substitutions in prM have the potential to increase infectivity in patients with pre-existing immunity. The prM substitution E228D, found in Clade C and its ancestors, is in the highly conserved N-terminus of the a-helical domain. Previous studies have shown that position 228 is involved in prM-E interactions (Hsieh et al. 2014), and we hypothesize that the change from glutamic acid to aspartic acid may alter the binding kinetics for prM and E, which could impact prM cleavage by furin and entry of virions into cells.

We also identified clade-specific amino acid substitutions in the envelope protein. The envelope protein contains three structural domains (DI-DIII) and a transmembrane anchor, which is involved in targeting it to sites of virion assembly (Klein et al. 2013). D-I organizes the structure of mature dimers, D-II consists of the dimerization domain and fusion loop, and D-III consists of the Ig-like receptor-binding region involved in viral entry (Kuhn et al. 2002, Modis et al. 2004). In Clade C, we identified a DII substitution at L478I in sequences from 2021 but not 2019. Interestingly, this substitution is predicted to increase hydrophobicity, and previous studies have shown that substitutions that increase hydrophobicity in DII contribute to immune escape (Messer et al. 2014). Finally, Clades C and B shared a common substitution (I737V) with sequences from Santander in 2001. This position is in the first α-helix of the transmembrane domain, which is primarily involved in the assembly of virus particles, so substitutions here may affect virion assembly (Hsieh et al. 2010).

In addition to mutations in structural proteins, we also identified several amino acid substitutions in NS1, including one site (S1068N) under positive selection. Interestingly, Salvo *et al*. also reported positive selection in NS1 among DENV-1 sequences from Colombia, though not at this specific position (Salvo et al. 2019). NS1 is a multi-function protein with two states: a membrane-associated dimer involved in intracellular viral replication and an oligomeric secreted form. The secreted form is linked to increased viral pathogenesis through the induction of endothelial dysfunction, vascular leak, and pro-inflammatory cytokine release from immune cells (Valero et al. 2014, Beatty et al. 2015, Modhiran et al. 2015, Glasner et al. 2017, Puerta-Guardo et al. 2019). The NS1 protein comprises an N-terminal B-roll, a Wing domain (surrounded by connector domains), and a β-Ladder domain (Akey et al. 2014). Previous studies have shown that antibodies targeting either the wing or β-ladder domains can block NS1 binding to endothelial or immune cells and are protective in lethal challenge models (Beatty et al. 2015, Brault et al. 2017, Lai et al. 2017, Bailey et al. 2018, Bailey, 2019, Espinosa et al. 2019, Biering et al. 2021, Modhiran et al. 2021, Wessel et al. 2021). Recently, highly protective NS1 antibodies have been mapped to conserved regions in the NS1 β-ladder domain that are directly adjacent to the S1068N mutation we identified (R1074 and T1076) (Biering et al. 2021). Therefore, we hypothesize that this substitution in the β-ladder may be involved in immune escape through glycosylation alterations or steric hindrance.

Overall, analysis of the sequences generated in this study revealed that contemporary DENV-1V viruses in Colombia descended from viruses that have been circulating within the country over the last 15 years. Despite recent increases in disease burden, we did not find evidence for the introduction of new lineages or sublineages to the country, but rather spread and diversification of DENV-1 within territories inside Colombia. We also observed co-circulation of multiple DENV-1V clades in nearby geographic regions, as well as clade-specific mutations, some of which may have a role in immune evasion or pathogenesis. Our study underscores the importance of understanding DENV evolution and dispersion in endemic regions.

## Supplementary Material

veaf018_Supp

## Data Availability

The sequences generated in this study have been deposited in the NCBI GenBank database. These sequences are publicly available to the scientific community under the accessions reported in supplementary material table S3. In addition, the corresponding Sequence Read Archive (SRA) has been uploaded and is accessible in the NCBI SRA database. These resources allow the reproduction of the analyses carried out and facilitate future studies.
