# Supplementary material for: Spatiotemporal dispersion of DENV-1 genotype V in Western Colombia
Source: Virus Evol. 2025 Apr 16;11(1):veaf018. doi: 10.1093/ve/veaf018 (PMC12091148; doi:10.1093/ve/veaf018)
Supplement: veaf018_Supp [file veaf018_supp.zip › suppl_data/Rojas-Gallardo_revised_supplemental_tables_FINAL.docx]

**Table S1. Primers for DENV-1 V multiplex amplicon sequencing**

| **Primer Name** | **Pool** | **DENV-specific primer (5'-3')** | **Sequence (with iNEXT fusions primers)**  **5'->3'** | **Length** | **Tm** | **Final Primer Concentration (µm)** |
| --- | --- | --- | --- | --- | --- | --- |
| DENV1_1_LEFT | 1 | GAGCAGATCTCTGATGAACAACCA | TCGTCGGCAGCGTCAGATGTGTATAAGAGACAGGAGCAGATCTCTGATGAACAACCA | 57 | 71.5 | 0.56 |
| DENV1_1_RIGHT | 1 | CATGTGTGGCTCTCCCCC | GTCTCGTGGGCTCGGAGATGTGTATAAGAGACAGCATGTGTGGCTCTCCCCC | 52 | 73 | 0.56 |
| DENV1_2_LEFT | 2 | TGACCATGCTCCTCATGCTG | TCGTCGGCAGCGTCAGATGTGTATAAGAGACAGTGACCATGCTCCTCATGCTG | 53 | 72.1 | 0.28 |
| DENV1_2_RIGHT | 2 | CCCAGGTCTCCACTCTTTGTATTT | GTCTCGTGGGCTCGGAGATGTGTATAAGAGACAGCCCAGGTCTCCACTCTTTGTATTT | 58 | 71.3 | 0.28 |
| DENV1_3_LEFT | 1 | ACACGTGGGACTTGGTCTAGAA | TCGTCGGCAGCGTCAGATGTGTATAAGAGACAGACACGTGGGACTTGGTCTAGAA | 55 | 71.7 | 0.56 |
| DENV1_3_RIGHT | 1 | TTGATATTTTAGCTTCAATGCACAGTTTG | GTCTCGTGGGCTCGGAGATGTGTATAAGAGACAGTTGATATTTTAGCTTCAATGCACAGTTTG | 63 | 76.6 | 0.56 |
| DENV1_4_LEFT | 2 | CGTCACCACCATGGCAAAAA | TCGTCGGCAGCGTCAGATGTGTATAAGAGACAGCGTCACCACCATGGCAAAAA | 53 | 72.6 | 0.42 |
| DENV1_4_RIGHT | 2 | GAGCTTGAGGTGTTATGGTTGC | GTCTCGTGGGCTCGGAGATGTGTATAAGAGACAGGAGCTTGAGGTGTTATGGTTGC | 56 | 71.4 | 0.42 |
| DENV1_5_LEFT | 1 | ATAGTCACTGTCCACACTGGGG | TCGTCGGCAGCGTCAGATGTGTATAAGAGACAGATAGTCACTGTCCACACTGGGG | 55 | 71.9 | 0.56 |
| DENV1_5_RIGHT | 1 | CCTGACGTCTGGATTTCTGTCG | GTCTCGTGGGCTCGGAGATGTGTATAAGAGACAGCCTGACGTCTGGATTTCTGTCG | 56 | 72 | 0.56 |
| DENV1_6_LEFT | 2 | ATCTGCTGGTCACATTCAAGAC | TCGTCGGCAGCGTCAGATGTGTATAAGAGACAGATCTGCTGGTCACATTCAAGAC | 55 | 70.8 | 1.13 |
| DENV1_6_RIGHT | 2 | TGTTGACTGGTTTCTCTTTGTCAGT | GTCTCGTGGGCTCGGAGATGTGTATAAGAGACAGTGTTGACTGGTTTCTCTTTGTCAGT | 59 | 70.6 | 1.13 |
| DENV1_7_LEFT | 1 | GCCATGCAAGATCCCCTTCT | TCGTCGGCAGCGTCAGATGTGTATAAGAGACAGGCCATGCAAGATCCCCTTCT | 53 | 72.7 | 0.56 |
| DENV1_7_RIGHT | 1 | CCATGTCAACAGAATCCCTATTCCT | GTCTCGTGGGCTCGGAGATGTGTATAAGAGACAGCCATGTCAACAGAATCCCTATTCCT | 59 | 71.1 | 0.56 |
| DENV1_8_LEFT | 2 | CATCTGTGGGAAAATTGGTACACC | TCGTCGGCAGCGTCAGATGTGTATAAGAGACAGCATCTGTGGGAAAATTGGTACACC | 57 | 71.5 | 0.7 |
| DENV1_8_RIGHT | 2 | TTCAATTCATTTGATATTTGCTTCCACAT | GTCTCGTGGGCTCGGAGATGTGTATAAGAGACAGTTCAATTCATTTGATATTTGCTTCCACAT | 63 | 75.9 | 0.7 |
| DENV1_9_LEFT | 1 | TGTCAGCAGCCATTGGAAAGG | TCGTCGGCAGCGTCAGATGTGTATAAGAGACAGTGTCAGCAGCCATTGGAAAGG | 54 | 72.2 | 0.56 |
| DENV1_9_RIGHT | 1 | GGGTGTAGGAGTCACGCAATTT | GTCTCGTGGGCTCGGAGATGTGTATAAGAGACAGGGGTGTAGGAGTCACGCAATTT | 56 | 71.9 | 0.56 |
| DENV1_10_LEFT | 2 | GACGGCCAAAGAGCATGGAA | TCGTCGGCAGCGTCAGATGTGTATAAGAGACAGGACGGCCAAAGAGCATGGAA | 53 | 72.9 | 0.56 |
| DENV1_10_RIGHT | 2 | TGGTGCCTTCACACAAGTCAAA | GTCTCGTGGGCTCGGAGATGTGTATAAGAGACAGTGGTGCCTTCACACAAGTCAAA | 56 | 71.4 | 0.56 |
| DENV1_11_LEFT | 1 | AGCACAACTACAGACCAGGGTA | TCGTCGGCAGCGTCAGATGTGTATAAGAGACAGAGCACAACTACAGACCAGGGTA | 55 | 71.7 | 0.7 |
| DENV1_11_RIGHT | 1 | AGTCATCAGCATCTTTCTACTCC | GTCTCGTGGGCTCGGAGATGTGTATAAGAGACAGAGTCATCAGCATCTTTCTACTCC | 57 | 69.8 | 0.7 |
| DENV1_12_LEFT | 2 | GGAGAAGTGGACAGTTTTTCATTAGG | TCGTCGGCAGCGTCAGATGTGTATAAGAGACAGGGAGAAGTGGACAGTTTTTCATTAGG | 59 | 70.7 | 0.62 |
| DENV1_12_RIGHT | 2 | GTCAACAATTTTAACATCATGATACCCA | GTCTCGTGGGCTCGGAGATGTGTATAAGAGACAGGTCAACAATTTTAACATCATGATACCCA | 62 | 76.7 | 0.62 |
| DENV1_13_LEFT | 1 | TGGTGGCATCCGTGGAG | TCGTCGGCAGCGTCAGATGTGTATAAGAGACAGTGGTGGCATCCGTGGAG | 50 | 72.7 | 0.62 |
| DENV1_13_RIGHT | 1 | GGAGTGAACTTAGTAGAATGCTGACT | GTCTCGTGGGCTCGGAGATGTGTATAAGAGACAGGGAGTGAACTTAGTAGAATGCTGACT | 60 | 70.5 | 0.62 |
| DENV1_14_LEFT | 2 | CAGAAAACAAAATCTGGGGAAGGA | TCGTCGGCAGCGTCAGATGTGTATAAGAGACAGCAGAAAACAAAATCTGGGGAAGGA | 57 | 71.3 | 0.84 |
| DENV1_14_RIGHT | 2 | TGCCAAAAATACCACACAAAAAGAGT | GTCTCGTGGGCTCGGAGATGTGTATAAGAGACAGTGCCAAAAATACCACACAAAAAGAGT | 60 | 70.2 | 0.84 |
| DENV1_15_LEFT | 1 | GACACACTTACTATACTCCTTAAAGC | TCGTCGGCAGCGTCAGATGTGTATAAGAGACAGGACACACTTACTATACTCCTTAAAGC | 59 | 69.5 | 1.13 |
| DENV1_15_RIGHT | 1 | TCAACAGCTATCACCTGCACTTC | GTCTCGTGGGCTCGGAGATGTGTATAAGAGACAGTCAACAGCTATCACCTGCACTTC | 57 | 71.1 | 1.13 |
| DENV1_16_LEFT | 2 | GGGCCAGTGTCAAAAAGGACTT | TCGTCGGCAGCGTCAGATGTGTATAAGAGACAGGGGCCAGTGTCAAAAAGGACTT | 55 | 72.4 | 0.84 |
| DENV1_16_RIGHT | 2 | TCTTCTTGTTTTTCCGGATCCTGG | GTCTCGTGGGCTCGGAGATGTGTATAAGAGACAGTCTTCTTGTTTTTCCGGATCCTGG | 58 | 70.9 | 0.84 |
| DENV1_17_LEFT | 1 | ATTGCCCAAGCTAAAGCATCACA | TCGTCGGCAGCGTCAGATGTGTATAAGAGACAGATTGCCCAAGCTAAAGCATCACA | 56 | 71.6 | 0.56 |
| DENV1_17_RIGHT | 1 | CTGGCCGCTATGCTGGC | GTCTCGTGGGCTCGGAGATGTGTATAAGAGACAGCTGGCCGCTATGCTGGC | 51 | 73.7 | 0.56 |
| DENV1_18_LEFT | 2 | TTATCCCCAGTGAGAGTTCC | TCGTCGGCAGCGTCAGATGTGTATAAGAGACAGTTATCCCCAGTGAGAGTTCC | 53 | 70.7 | 0.84 |
| DENV1_18_RIGHT | 2 | TGTTGTGACAACATAATCCCAGTC | GTCTCGTGGGCTCGGAGATGTGTATAAGAGACAGTGTTGTGACAACATAATCCCAGTC | 58 | 70.2 | 0.84 |
| DENV1_19_LEFT | 1 | GAAAACGGGTAATCCAATTGAGCA | TCGTCGGCAGCGTCAGATGTGTATAAGAGACAGGAAAACGGGTAATCCAATTGAGCA | 57 | 71.3 | 0.56 |
| DENV1_19_RIGHT | 1 | TCTCTCTCTGGCTCAAAGAGGG | GTCTCGTGGGCTCGGAGATGTGTATAAGAGACAGTCTCTCTCTGGCTCAAAGAGGG | 56 | 71.5 | 0.56 |
| DENV1_20_LEFT | 2 | GGATCACGCTCATTGGACAGAA | TCGTCGGCAGCGTCAGATGTGTATAAGAGACAGGGATCACGCTCATTGGACAGAA | 55 | 72.1 | 0.42 |
| DENV1_20_RIGHT | 2 | GACACTTCTTCTTCCTGCTGCA | GTCTCGTGGGCTCGGAGATGTGTATAAGAGACAGGACACTTCTTCTTCCTGCTGCA | 56 | 71.8 | 0.42 |
| DENV1_21_LEFT | 1 | CTGGACAAAGGAAGGAGAAAGAAAGA | TCGTCGGCAGCGTCAGATGTGTATAAGAGACAGCTGGACAAAGGAAGGAGAAAGAAAGA | 59 | 71.3 | 0.7 |
| DENV1_21_RIGHT | 1 | CCATCCATAACAGTGCGCTTGA | GTCTCGTGGGCTCGGAGATGTGTATAAGAGACAGCCATCCATAACAGTGCGCTTGA | 56 | 72.2 | 0.7 |
| DENV1_22_LEFT | 2 | GGTGGAGTGACGCTATTCTTCC | TCGTCGGCAGCGTCAGATGTGTATAAGAGACAGGGTGGAGTGACGCTATTCTTCC | 55 | 72.1 | 0.28 |
| DENV1_22_RIGHT | 2 | TGGGAGTGATAACTGTTGTGGC | GTCTCGTGGGCTCGGAGATGTGTATAAGAGACAGTGGGAGTGATAACTGTTGTGGC | 56 | 71.3 | 0.28 |
| DENV1_23_LEFT | 1 | CACCAACATGCTACAATGCTGG | TCGTCGGCAGCGTCAGATGTGTATAAGAGACAGCACCAACATGCTACAATGCTGG | 55 | 72.2 | 0.34 |
| DENV1_23_RIGHT | 1 | TCTATTGCAACAATCCCGTCTACG | GTCTCGTGGGCTCGGAGATGTGTATAAGAGACAGTCTATTGCAACAATCCCGTCTACG | 58 | 70.8 | 0.34 |
| DENV1_24_LEFT | 2 | AAGCAAAGGCCACTAGAGAAGC | TCGTCGGCAGCGTCAGATGTGTATAAGAGACAGAAGCAAAGGCCACTAGAGAAGC | 55 | 71.6 | 0.28 |
| DENV1_24_RIGHT | 2 | TTTCTCTCCCAGTGTTTCCCCT | GTCTCGTGGGCTCGGAGATGTGTATAAGAGACAGTTTCTCTCCCAGTGTTTCCCCT | 56 | 71.6 | 0.28 |
| DENV1_25_LEFT | 1 | GGAGCAGGTCTGGCTTTTTCAT | TCGTCGGCAGCGTCAGATGTGTATAAGAGACAGGGAGCAGGTCTGGCTTTTTCAT | 55 | 72.4 | 0.56 |
| DENV1_25_RIGHT | 1 | CCATAGGTCGCCATTGGGAT | GTCTCGTGGGCTCGGAGATGTGTATAAGAGACAGCCATAGGTCGCCATTGGGAT | 54 | 72.3 | 0.56 |
| DENV1_26_LEFT | 2 | TGCTGGGCTGAAGAAAGTTACT | TCGTCGGCAGCGTCAGATGTGTATAAGAGACAGTGCTGGGCTGAAGAAAGTTACT | 55 | 71.4 | 0.48 |
| DENV1_26_RIGHT | 2 | TTTCCTGTTCCACATGAAACCCA | GTCTCGTGGGCTCGGAGATGTGTATAAGAGACAGTTTCCTGTTCCACATGAAACCCA | 57 | 71.1 | 0.48 |
| DENV1_27_LEFT | 1 | AAACACGGAGGGATGCTAGTG | TCGTCGGCAGCGTCAGATGTGTATAAGAGACAGAAACACGGAGGGATGCTAGTG | 54 | 71.6 | 0.56 |
| DENV1_27_RIGHT | 1 | ACATCCCATGGTTTTGTGAGCA | GTCTCGTGGGCTCGGAGATGTGTATAAGAGACAGACATCCCATGGTTTTGTGAGCA | 56 | 71.3 | 0.56 |
| DENV1_28_LEFT | 2 | CATGGATCATATGAGGTCAAGCCA | TCGTCGGCAGCGTCAGATGTGTATAAGAGACAGCATGGATCATATGAGGTCAAGCCA | 57 | 71.7 | 0.28 |
| DENV1_28_RIGHT | 2 | TCTCTGTGCACGAGATCCCA | GTCTCGTGGGCTCGGAGATGTGTATAAGAGACAGTCTCTGTGCACGAGATCCCA | 54 | 71.9 | 0.28 |
| DENV1_29_LEFT | 1 | AGGAGCAGTGTTCGTTGATGAAA | TCGTCGGCAGCGTCAGATGTGTATAAGAGACAGAGGAGCAGTGTTCGTTGATGAAA | 56 | 71.3 | 0.56 |
| DENV1_29_RIGHT | 1 | TTATTCTTGTGTCCCATCCGGC | GTCTCGTGGGCTCGGAGATGTGTATAAGAGACAGTTATTCTTGTGTCCCATCCGGC | 56 | 71.3 | 0.56 |
| DENV1_30_LEFT | 2 | GGACTGCACAAACTTGGATACA | TCGTCGGCAGCGTCAGATGTGTATAAGAGACAGGGACTGCACAAACTTGGATACA | 55 | 71.4 | 0.7 |
| DENV1_30_RIGHT | 2 | CCAACCAGTCAAGAACTCTCTCAG | GTCTCGTGGGCTCGGAGATGTGTATAAGAGACAGCCAACCAGTCAAGAACTCTCTCAG | 58 | 71.4 | 0.7 |
| DENV1_31_LEFT | 1 | GGAGGTCCAACTAATAAGACAAATGGA | TCGTCGGCAGCGTCAGATGTGTATAAGAGACAGGGAGGTCCAACTAATAAGACAAATGGA | 60 | 70.8 | 0.56 |
| DENV1_31_RIGHT | 1 | AGTTTCTCTCAGGCTCCATCC | GTCTCGTGGGCTCGGAGATGTGTATAAGAGACAGAGTTTCTCTCAGGCTCCATCC | 55 | 71 | 0.56 |
| DENV1_32_LEFT | 2 | AGGGAAATAGTGGTGCCATGC | TCGTCGGCAGCGTCAGATGTGTATAAGAGACAGAGGGAAATAGTGGTGCCATGC | 54 | 71.9 | 0.42 |
| DENV1_32_RIGHT | 2 | TGTCAAGCCTATCAGGGATCCA | GTCTCGTGGGCTCGGAGATGTGTATAAGAGACAGTGTCAAGCCTATCAGGGATCCA | 56 | 71.4 | 0.42 |
| DENV1_33_LEFT | 1 | GGATGGAGGACAAAACTCATGTATCC | TCGTCGGCAGCGTCAGATGTGTATAAGAGACAGGGATGGAGGACAAAACTCATGTATCC | 59 | 71.3 | 0.56 |
| DENV1_33_RIGHT | 1 | CTTGCTCAATCCGTGGCTTTC | GTCTCGTGGGCTCGGAGATGTGTATAAGAGACAGCTTGCTCAATCCGTGGCTTTC | 55 | 72 | 0.56 |
| DENV1_34_LEFT | 2 | CGAAGGAGCACTCTGGTAAGTC | TCGTCGGCAGCGTCAGATGTGTATAAGAGACAGCGAAGGAGCACTCTGGTAAGTC | 55 | 72.2 | 0.65 |
| DENV1_34_RIGHT | 2 | TGGTCTCTCCCAGCGTCAATAT | GTCTCGTGGGCTCGGAGATGTGTATAAGAGACAGTGGTCTCTCCCAGCGTCAATAT | 56 | 71.5 | 0.65 |

**Table S2.** **Clinical and laboratory-confirmed DENV cases in two Colombian departments: Valle del Cauca and Risaralda**. Serum from patients with acute febrile symptoms (Risaralda) and clinically diagnosed DENV (Valle del Cauca) were analyzed using the NS1/IgM/IgG rapid test and RT-qPCR. Samples that were positive for DENV RNA underwent typing RT-qPCR, and DENV-1 samples underwent full genome sequencing.

| **Department** | **Samples** | **NS1/IgM rapid test** | **RT-qPCR** | **Serotype** | | | | **Sequenced** |
| --- | --- | --- | --- | --- | --- | --- | --- | --- |
|  |  |  |  | **DENV1** | **DENV2** | **DENV3** | **DENV4** |  |
| Valle del Cauca | 201 | 96 | 79 | 42 | 20 | 4 | 0 | 17 |
| Risaralda | 178 | 20 | 20 | 7 | 1 | 0 | 0 | 7 |

**Table S3.  Twenty-four new full-length DENV1 genotype V sequences were generated from two Colombian departments from 2019-2022**.

| GenBank Accession | Department | Collection Date | Sample ID | Coverage |
| --- | --- | --- | --- | --- |
| PP957577 | Valle del Cauca | 8/15/2021 | VAC_DFC059_2021 | 100% |
| PP957578 | Valle del Cauca | 8/15/2021 | VAC_DFC063_2021 | 100% |
| PP957579 | Valle del Cauca | 8/24/2021 | VAC_DFC087_2021 | 100% |
| PP957580 | Valle del Cauca | 8/28/2021 | VAC_DFC088_2021 | 100% |
| PP957581 | Valle del Cauca | 8/30/2021 | VAC_DFC096_2021 | 100% |
| PP957582 | Valle del Cauca | 9/6/2021 | VAC_DFC112_2021 | 100% |
| PP957583 | Valle del Cauca | 9/6/2021 | VAC_DFC114_2021 | 100% |
| PP957584 | Valle del Cauca | 10/16/2021 | VAC_DFC161_2021 | 100% |
| PP957585 | Valle del Cauca | 10/17/2021 | VAC_DFC162_2021 | 100% |
| PP957586 | Valle del Cauca | 10/29/2021 | VAC_DFC177_2021 | 100% |
| PP957591 | Valle del Cauca | 5/11/2022 | VAC_DFC263_2022 | 100% |
| PP957592 | Valle del Cauca | 11/11/2022 | VAC_DFC264_2022 | 100% |
| PP957593 | Valle del Cauca | 11/12/2022 | VAC_DFC265_2022 | 99% |
| PP957590 | Valle del Cauca | 7/24/2022 | VAC_DFC245_2022 | 100% |
| PP957589 | Valle del Cauca | 12/12/2021 | VAC_DFC238_2021 | 100% |
| PP957588 | Valle del Cauca | 12/3/2021 | VAC_DFC222_2021 | 100% |
| PP957587 | Valle del Cauca | 11/30/2021 | VAC_DFC217_2021 | 100% |
| PP957573 | Risaralda | 3/3/2021 | RIS_AFI 031_2021 | 100% |
| PP957574 | Risaralda | 6/28/2021 | RIS_AFI 082_2021 | 95% |
| PP957575 | Risaralda | 8/5/2021 | RIS_AFI 101_2021 | 96% |
| PP957576 | Risaralda | 4/21/2022 | RIS_AFI 224_2022 | 100% |
| OM654348 | Risaralda | 1/25/2021 | RIS_OM654348_202 | 100% |
| MZ773407 | Risaralda | 9/21/2019 | RIS_MZ773407_2019 | 100% |
| OM654347 | Risaralda | 1/25/2021 | RIS_OM654347_202 | 100% |

**Table S4.** Nested sampling results for model selection for CDS and envelope data sets

| **CDS genetic proximity-subsampled data set** | | | | |
| --- | --- | --- | --- | --- |
| **Model** | **Marginal Likelihood** | **SD** | **Information** | **ML Difference** |
| Strict_Constant | -43656.55606 | sqrt(H/N)=(2.0)=?=SD=(1.9) | 267.6 | 310.8 |
| Strict _Exponential | -43792.87992 | sqrt(H/N)=(2.0)=?=SD=(2.0) | 269.1 | 447.1 |
| Strict _BS | -43662.96482 | sqrt(H/N)=(1.9)=?=SD=(1.9) | 268.8 | 317.2 |
| Strict _EBS | -43658.8195 | sqrt(H/N)=(2.0)=?=SD=(2.0) | 272.8 | 313.0 |
| Relax Lognormal_Constant | -43361.37548 | sqrt(H/N)=(2.0)=?=SD=(2.0) | 284.8 | 15.6 |
| Relax Lognormal_Exponential | -43377.10127 | sqrt(H/N)=(2.0)=?=SD=(2.0) | 284.8 | 31.3 |
| Relax Lognormal_BS* | -43345.78699 | sqrt(H/N)=(2.0)=?=SD=(2.0) | 284.5 |  |
| Relax Lognormal_EBS | -43359.01884 | sqrt(H/N)=(2.0)=?=SD=(1.9) | 284.6 | 13.2 |
| **Envelope genetic proximity-subsampled data set** | | | | |
| **Model** | **Marginal Likelihood** | **SD** | **Information** | **ML Difference** |
| Strict_Constant | -8516.409458 | sqrt(H/N)=(8.4)=?=SD=(8.3) | 1119.2 | 46.7 |
| Strict _Exponential | -8530.255461 | sqrt(H/N)=(8.3)=?=SD=(8.5) | 1104.8 | 60.5 |
| Strict _BS* | -8469.750158 | sqrt(H/N)=(8.2)=?=SD=(8.2) | 1097.7 |  |
| Strict _EBS | -8515.660103 | sqrt(H/N)=(8.4)=?=SD=(8.6) | 1141.3 | 45.9 |
| Relax Lognormal_Constant | -8517.495131 | sqrt(H/N)=(8.5)=?=SD=(8.6) | 1146.1 | 47.7 |
| Relax Lognormal_Exponential | -8509.69687 | sqrt(H/N)=(8.6)=?=SD=(9.1) | 1192.2 | 39.9 |
| Relax Lognormal_BS | -8496.329628 | sqrt(H/N)=(8.6)=?=SD=(8.4) | 1193.8 | 26.6 |
| Relax Lognormal_EBS | -8737.887388 | sqrt(H/N)=(8.8)=?=SD=(9.0) | 1228.2 | 268.1 |
| **Envelope genetic proximity-subsampled data set, restricted to the smallest clade containing our new sequences** | | | | |
| **Model** | **Marginal Likelihood** | **SD** | **Information** | **ML Difference** |
| Strict_Constant | -8436.322628 | sqrt(H/N)=(8.5)=?=SD=(8.9) | 1083.8 | ML Diference |
| Strict _Exponential | -8477.296131 | sqrt(H/N)=(8.4)=?=SD=(8.7) | 1046.0 | 47.4721705 |
| Strict _BS* | -8481.69687 | sqrt(H/N)=(8.7)=?=SD=(9.0) | 1102.2 | 88.4456736 |
| Relax Lognormal_Constant | -8388.850458 | sqrt(H/N)=(8.3)=?=SD=(8.1) | 1097.7 | 92.8464121 |
| Relax Lognormal_Exponential | -8476.924472 | sqrt(H/N)=(8.4)=?=SD=(8.2) | 1029.2 |  |
| Relax Lognormal_BS | -8570.359461 | sqrt(H/N)=(8.3)=?=SD=(8.6) | 1109.6 | 88.0740144 |
| **Envelope geography-subsampled data set** | | | | |
| **Model** | **Marginal Likelihood** | **SD** | **Information** | **ML Difference** |
| Strict_Constant | -31524.39567 | sqrt(H/N)=(5.89)=?=SD=(6.06) | 828.28 | 69.01 |
| Strict _Exponential | -31607.71135 | sqrt(H/N)=(5.89)=?=SD=(5.97) | 834.11 | 152.33 |
| Strict _BS* | -31455.38625 | sqrt(H/N)=(5.89)=?=SD=(6.13) | 834.31 |  |
| Relax Lognormal_Constant | -31582.42358 | sqrt(H/N)=(5.86)=?=SD=(6.01) | 915.32 | 127.04 |
| Relax Lognormal_Exponential | -31569.42563 | sqrt(H/N)=(5.91)=?=SD=(6.02) | 895.26 | 114.04 |
| Relax Lognormal_BS | -31545.85245 | sqrt(H/N)=(5.92)=?=SD=(6.01) | 847.45 | 90.47 |

*Model with the highest likelihood value. Minimum expected difference for CDS genetic proximity-subsampled data set: 5.7. Minimum expected difference for envelope genetic proximity-subsampled data set: 23.6. Minimum expected difference for envelope genetic proximity-subsampled data set, restricted to the smallest clade containing our new sequences: 24.0. Minimum expected difference for envelope geography-subsampled data set: 16.8.

**Table S5**. **Summary of the Time to the Most Recent Common Ancestor (TMRCA) for nodes of interest.** The CDS data was analyzed with a best fit model using a relaxed molecular clock, and the envelope gene dataset was analyzed with a best fit model using a strict molecular clock. NC=not calculated, since the CDS data set does not include the sequences from Santander used to identify clade B*.

| **Data set** | **Clade** | **MRCA date** | **HPDI** | |
| --- | --- | --- | --- | --- |
| ***CDS genetic proximity-subsampled*** | Clade A | 2017.88 | 2015.73 | 2019.84 |
|  | Clade A* | 2014.21 | 2012.78 | 2015.02 |
|  | Clade B | 2016.74 | 2015.01 | 2018.38 |
|  | Clade B* | NC | NC | NC |
|  | Clade C | 2018.78 | 2017.95 | 2019.21 |
|  | Clade C* | 2013.28 | 2012.51 | 2013.80 |
| ***Envelope genetic proximity-subsampled*** | Clade A | 2018.06 | 2016.42 | 2020.16 |
|  | Clade A* | 2013.85 | 2012.37 | 2015.35 |
|  | Clade B | 2018.11 | 2016.66 | 2019.75 |
|  | Clade B* | 2007.37 | 2006.51 | 2008.34 |
|  | Clade C | 2018.51 | 2017.43 | 2019.25 |
|  | Clade C* | 2021.32 | 2011.37 | 2013.50 |
| ***Envelope genetic proximity-subsampled*** *data set, restricted to the smallest clade containing our new sequences* | Clade A | 2018.07 | 2016.17 | 2019.89 |
|  | Clade A* | 2013.86 | 2012.12 | 2015.10 |
|  | Clade B | 2018.11 | 2016.40 | 2019.50 |
|  | Clade B* | 2007.59 | 2007.02 | 2008.23 |
|  | Clade C | 2018.52 | 2017.18 | 2019.25 |
|  | Clade C* | 2012.76 | 2011.78 | 2013.52 |
| ***Envelope geography-subsampled*** | Clade A | 2017.96 | 2016.14 | 2019.76 |
|  | Clade A* | 2013.87 | 2012.43 | 2015.07 |
|  | Clade B | 2018.08 | 2016.56 | 2019.46 |
|  | Clade B* | 2006.80 | 2004.66 | 2008.11 |
|  | Clade C | 2018.54 | 2017.48 | 2019.25 |
|  | Clade C* | 2012.81 | 2011.78 | 2013.63 |

**Table S6. Positive selection analysis for DENV-1V sequences.** Observed positions under positive selection in the envelope (n=300) and CDS (n=209) genetic proximity-subsampled datasets. Pervasive site-based models – Fixed Effects Likelihood (FEL), Single-Likelihood Ancestor Counting (SLAC), and Fast, Unconstrained Bayesian AppRoximation (FUBAR) – were used to identify positions under persistent selection. The adaptive site-based model, Mixed Effects Model of Evolution (MEME), was used to identify codons under episodic selection. The default settings were used for each model. The position of each codon is relative to the position in each coding region. Selection analysis was implemented using models from HyPhy.

| **Dataset** | **AA Position** | **Protein** | **MEME** (P-value) | **FEL** | **FUBAR** (probability) | **SLAC** |
| --- | --- | --- | --- | --- | --- | --- |
|  |  |  |  | (p-value) |  | (p-value) |
| *Envelope* | 248 | E | 0.02 | 0.01 | 1 | 0.07 |
| CDS | 1068 | NS1 | 0.03 | 0.02 | 0.99 | 0.23 |
