# Supplementary figures and images for: Spatiotemporal dispersion of DENV-1 genotype V in Western Colombia
Source: Virus Evol. 2025 Apr 16;11(1):veaf018. doi: 10.1093/ve/veaf018 (PMC12091148; doi:10.1093/ve/veaf018)

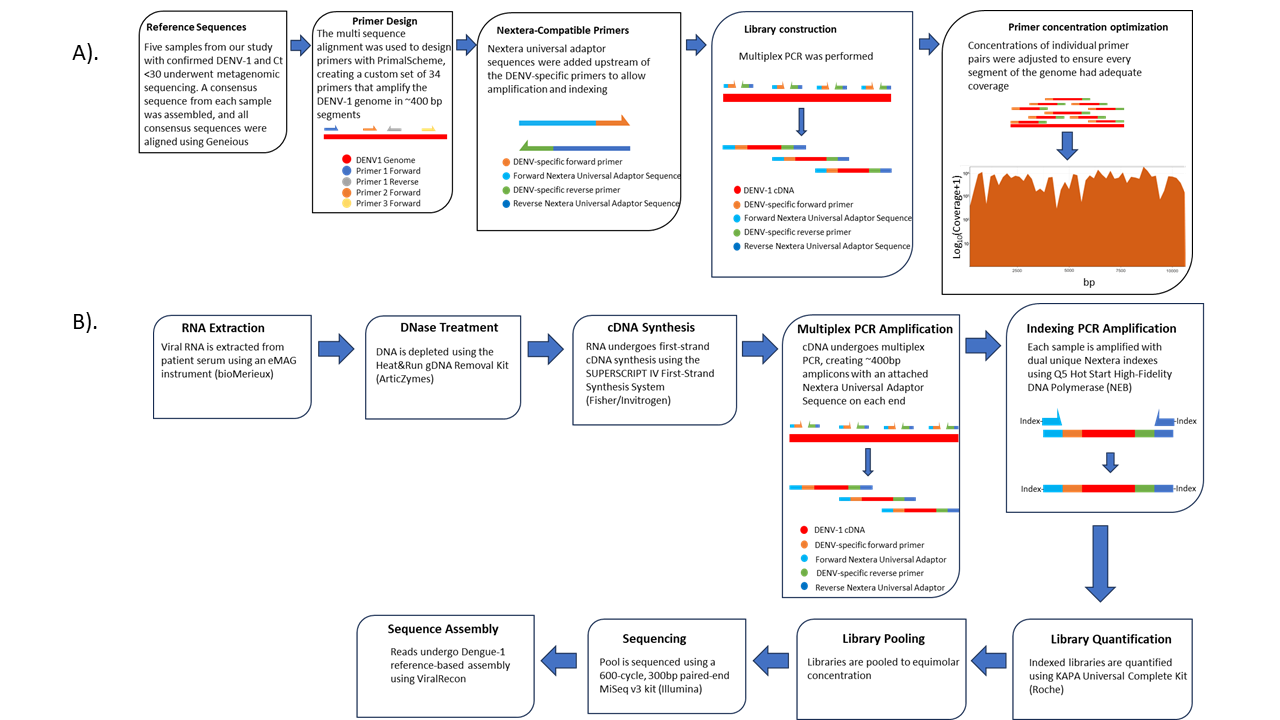

Supplement: veaf018_Supp [file veaf018_supp.zip › suppl_data/Figure S2.tif]

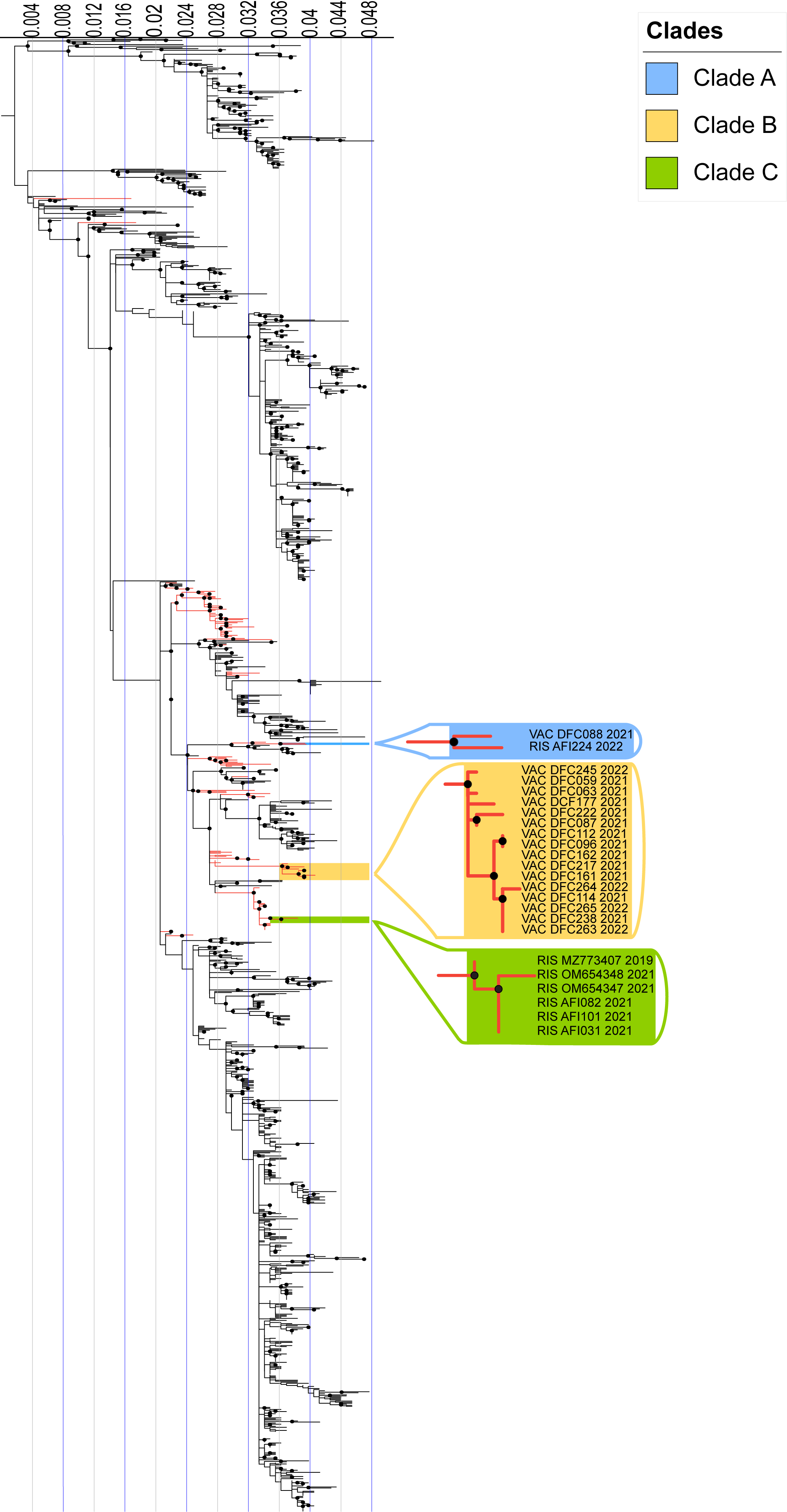

Supplement: veaf018_Supp [file veaf018_supp.zip › suppl_data/Figure S5.tiff]

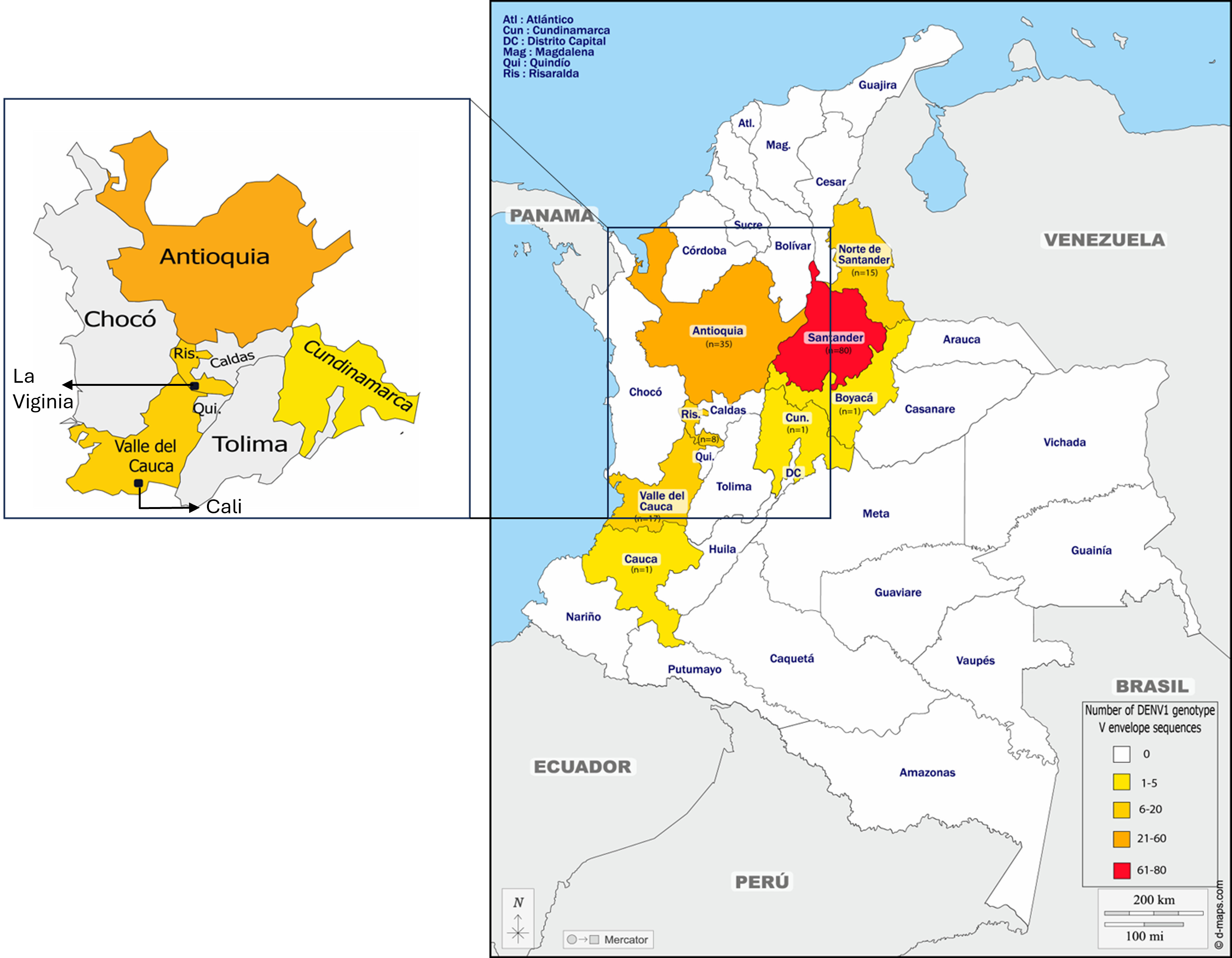

Supplement: veaf018_Supp [file veaf018_supp.zip › suppl_data/Figure_S1.tif]

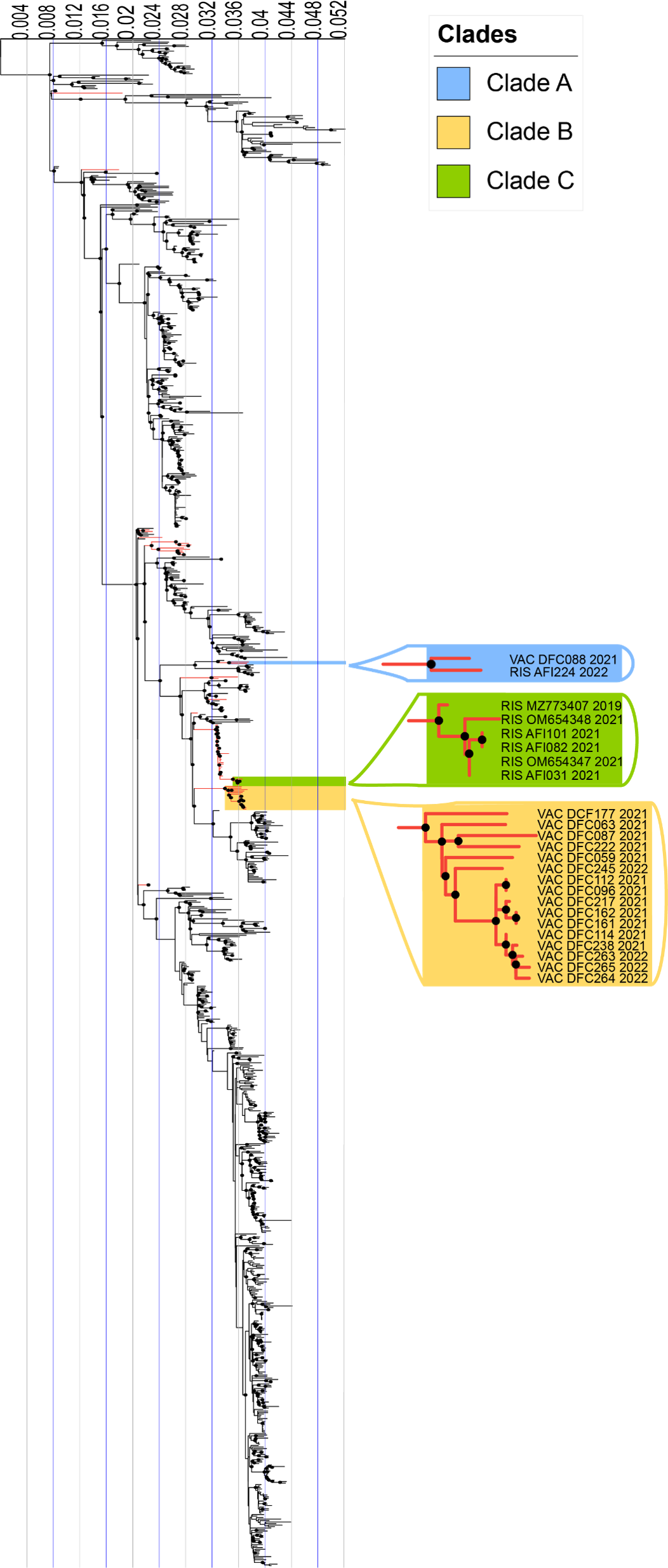

Supplement: veaf018_Supp [file veaf018_supp.zip › suppl_data/Figure_S3.tif]

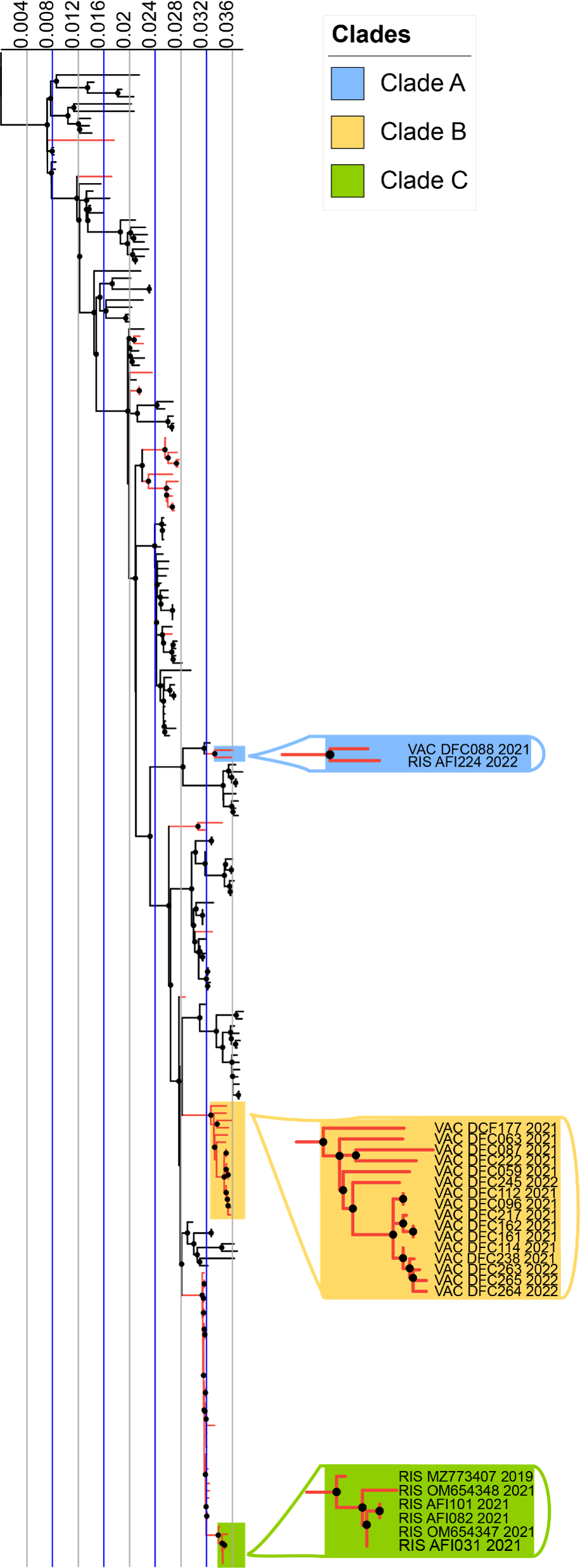

Supplement: veaf018_Supp [file veaf018_supp.zip › suppl_data/Figure_S4.tif]

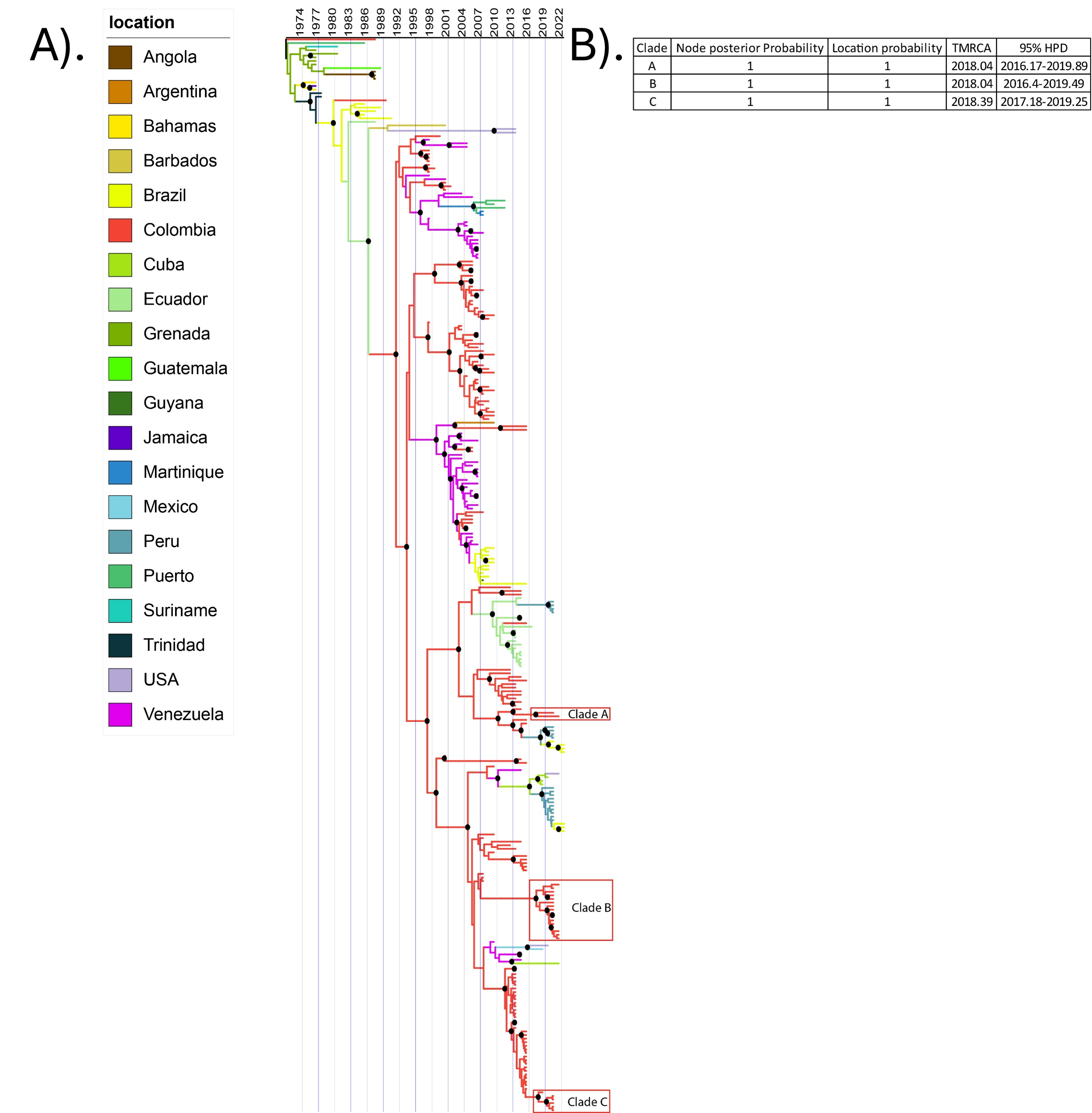

Supplement: veaf018_Supp [file veaf018_supp.zip › suppl_data/Figure_S6.tif]

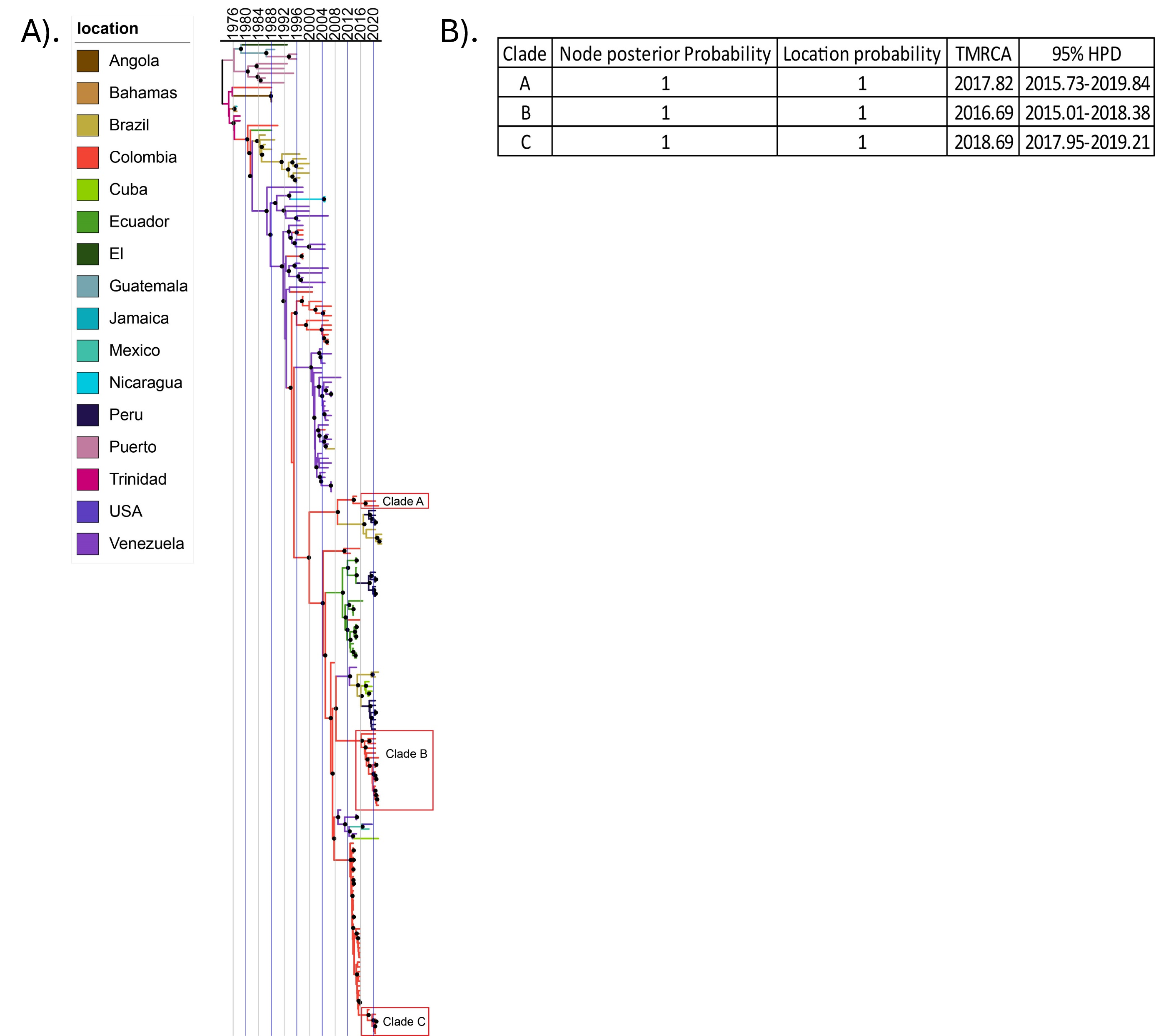

Supplement: veaf018_Supp [file veaf018_supp.zip › suppl_data/Figure_S7.tif]

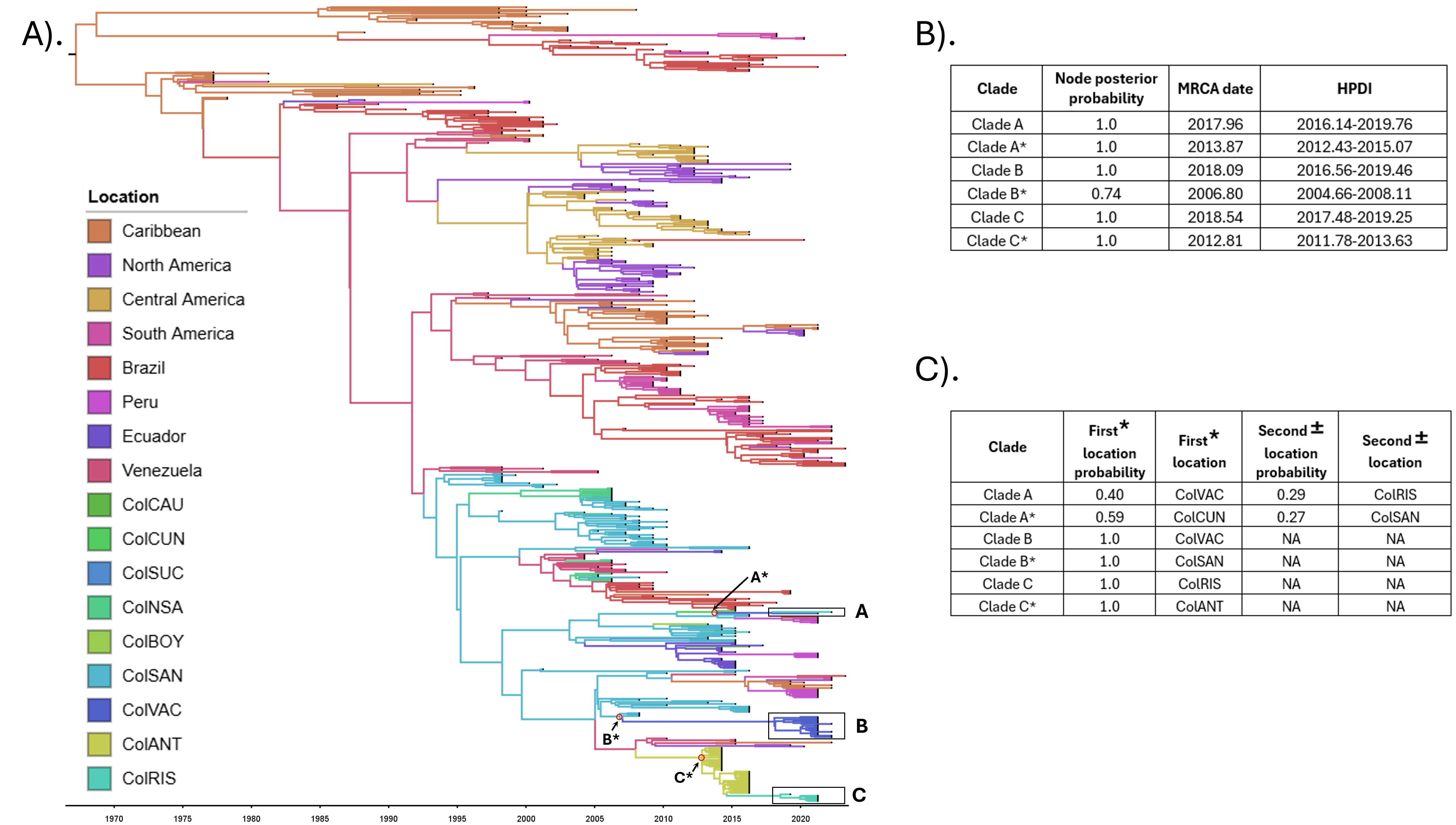

Supplement: veaf018_Supp [file veaf018_supp.zip › suppl_data/Figure_S8.tif]
